# COVID‐19, obesity, and immune response 2 years after the pandemic: A timeline of scientific advances

**DOI:** 10.1111/obr.13496

**Published:** 2022-07-15

**Authors:** Mayara Belchior‐Bezerra, Rafael Silva Lima, Nayara I. Medeiros, Juliana A. S. Gomes

**Affiliations:** ^1^ Laboratório de Biologia das Interações Celulares, Departamento de Morfologia, Instituto de Ciências Biológicas Universidade Federal de Minas Gerais Belo Horizonte Brazil; ^2^ Imunologia Celular e Molecular Instituto René Rachou, Fundação Oswaldo Cruz ‐ FIOCRUZ Belo Horizonte Brazil

**Keywords:** COVID‐19, immune response, obesity, scientific advances

## Abstract

In the 2 years since the COVID‐19 pandemic was officially declared, science has made considerable strides in understanding the disease's pathophysiology, pharmacological treatments, immune response, and vaccination, but there is still much room for further advances, especially in comprehending its relationship with obesity. Science has not yet described the mechanisms that explain how obesity is directly associated with a poor prognosis. This paper gathers all published studies over the past 2 years that have described immune response, obesity, and COVID‐19, a historical and chronological record for researchers and the general public alike. In summary, these studies describe how the cytokine/adipokine levels and inflammatory markers, such as the C‐reactive protein, are associated with a higher body mass index in COVID‐19‐positive patients, suggesting that the inflammatory background and immune dysregulation in individuals with obesity may be expressed in the results and that adiposity may influence the immune response. The timeline presented here is a compilation of the results of 2 years of scientific inquiry, describing how the science has progressed, the principal findings, and the challenges ahead regarding SARS‐CoV‐2, COVID‐19, and emerging variants, especially in patients with obesity.

## 1 INTRODUCTION

COVID‐19 is caused by SARS‐CoV‐2, an enveloped, single‐stranded, positive‐sense RNA virus that belongs to the Coronaviridae family. It may be asymptomatic or cause gastrointestinal, cardiovascular, and respiratory symptoms and may be potentially fatal.^1^ Moreover, in the course of the pandemic, several variants have surfaced with varying characteristic symptoms, degrees of transmissibility, and mortality rates.^2^, ^3^, ^4^ On December 31, 2019, the World Health Organization (WHO) noticed cases of viral “pneumonia of unknown origin” in the Chinese region of Wuhan. Shortly thereafter, the etiologic agent was found to be a coronavirus and its genome was sequenced. The disease name COVID‐19 was coined on February 11, 2020, and on March 11, the WHO declared COVID‐19 a pandemic due to the sharp rise in the number of cases and deaths worldwide.^5^, ^6^ To date, COVID‐19 has caused more than 400 million cases and over 6 million deaths, currently ranking as the seventh most lethal virus in human history.^7^ A global effort has been put forward to answer questions raised by the disease, including its origin, the impact on human and environmental health, and the best practices for treatment and prevention. As a result, exactly 2 years from the date of the first WHO report, a search using COVID‐19 as a keyword in PubMed retrieves 213,121 articles.

However, the immune response against COVID‐19 is not yet fully understood. Questions continue to puzzle immunologists, such as why some individuals are asymptomatic while others who seem to be otherwise healthy may have a severe case of the disease, how long immune memory can last after infection or vaccination, and how the immune system responds to the variants. Nevertheless, it is known that the immune system is involved in confronting the virus itself and the tissue damage that may occur. Briefly, the innate immune system's pathogen‐recognition receptors recognize RNA from the virus and trigger the inflammatory production of cytokines, such as interleukin 6 (IL‐6), tumour necrosis factor alpha, IL‐1, and type I and III interferons in particular. The adaptive immune response is involved when B and T lymphocytes are primed by viral antigens that bind to the B‐cell receptor and the T‐cell receptor, respectively. T cells can either help the development of the immune response and tissue repair by producing cytokines (CD4^+^ T cells) or by killing infected cells in an attempt to reduce the viral burden (CD8^+^ T cells), while B cells produce antibodies that endeavor to neutralize the infective potential of the virus.^8^, ^9^ The adaptive immune response is dampened after removing the threat, but T and B memory cells specific to the antigen are generated. As the evidence shows, immunological memory can last for several months with humoral – assessed by anti‐spike (S), anti‐nucleocapsid (N), and neutralizing antibodies – and cellular components – particularly memory B and T cells – exhibiting different kinetics in the peripheral blood.^10^, ^11^ Moreover, some form of memory may last years after infection, as suggested by data on antibodies in the context of SARS‐CoV infection.^12^ Nevertheless, reinfection may occur. While there is still no consensus in the literature as to the criterion used to define reinfection, some studies have shown that it is arguably rare, although emerging variants are likely to play an important role in this phenomenon, as reinfections are usually arise from strains from those of the first infection.^13^, ^14^, ^15^, ^16^ Importantly, vaccines seem to activate several pathways in the immune system, including the production of neutralizing and non‐neutralizing antibodies, induction of memory T and B cells, enhanced phagocytosis, and induction of type I and III interferons.^17^

An important feature of COVID‐19 is its higher rate of morbidity and mortality among individuals with comorbidities.^18^ Some systematic reviews and meta‐analyses have observed that patients with obesity have a higher risk of being hospitalized with a more severe case of the disease and dying.^19^, ^20^, ^21^ In addition to its elevated adipose tissue mass, obesity is characterized by low‐grade inflammation, or metaflammation, that affects the whole organism.^22^ In this context, there is an imbalance in the production of proinflammatory and anti‐inflammatory molecules. The adipose tissue also alters the release of its adipokines, which further contributes to the immune imbalance in obesity and changes the energy metabolism circuitry.^22^, ^23^ When combined with the metabolic burden imposed by obesity, along with a higher prevalence of pulmonary diseases, immune imbalance may be of prime importance to understanding why patients with obesity are especially vulnerable to COVID‐19.

The evidence of immune imbalance in severe COVID‐19 patients indicates a compromise in both the innate and adaptive immune systems.^24^ Obesity is also significantly associated with COVID‐19 complications, yet it is not well known how the immune system is affected by the double burden of obesity and COVID‐19 and whether vaccine protection is less effective in individuals with obesity. This scoping review aimed to answer the following question: What immunological advances have been made in the context of obesity as a comorbidity of COVID‐19 2 years after the first report by the WHO?

## 2 METHODS

This scoping review was conducted in accordance with the Preferred Reporting Items for Systematic Reviews and Meta‐Analyses Extension for Scoping Reviews. To answer the question posed in section 1, articles of all types were eligible if indexed in common search databases for medical literature, published in the English language between December 31, 2019 and December 31, 2021, and contained potential reports on how the immune system might be altered in obesity as a comorbidity of COVID‐19 and/or how the response after vaccination might differ due to changes in the BMI. PubMed, Web of Science, and Embase were used as databases for searching and retrieving eligible articles. Two filters – title and abstract – were used to select the articles that could help answer the question. Additional articles were retrieved from the references of the studies included in the scoping review.

The keywords and Medical Subject Headings were divided into three groups to include the largest number of articles in the stipulated period: COVID‐19, obesity, and immune system (Table 1). The chosen databases were then searched within the three groups, complying with the analysis strategies of each database and applying the described filters. Finally, a new search was performed combining the three groups, with the articles containing one word from each group.

**TABLE 1:** Research strategy groups

GROUP 1: COVID‐19	COVID‐19, Severe acute respiratory syndrome coronavirus 2, Severe Acute respiratory syndrome, 2019ncov, 2019 ncov, Novel coronavirus, Novel corona virus, Covid19, Severe acute respiratory syndrome coronavirus, Severe acute respiratory syndrome virus, and Severe acute respiratory infection
GROUP 2: OBESITY	Obesity, Childhood obesity, Adipos*, Adipose tissue, Types of adipose tissue, White adipose tissue, Brown adipose tissue, Beige adipose tissue, Visceral adipose tissue, Subcutaneous adipose tissue, Adipose tissue macrophage, Adipocytes, Pre‐adipocytes, Adipocytes, Fatty acids
GROUP 3: IMMUNE SYSTEM	Monocyte, Neutrophil, Basophil, Lymphocytes, NK, Natural killer cell, Eosinophil, Mast cell, ILC, Innate Lymphoid Cell, Dendritic cell, APC cell, Memory cell, Cytokines, Chemokine, Innate Immune System, Adaptive immune system, Antibodies, Vaccines, Macrophage, CD, Receptor, Covid‐19 Vaccine, Vaccine, Adjuvant, Efficacy Rate, Immunization, Recombinant, Side Effect, Vaccination, and Waning Immunity

All articles were screened by two independent researchers for each database, then downloaded and annotated, and the full text was read. Included were articles published between December 31, 2019 and December 31, 2021, with their full text in English, and with their title and/or abstracts referencing at least one word from each of the three groups. The criteria for exclusion were reviews; letters to the editor; editorials; comments; articles that did not add new results to the research question; articles that did not explicitly refer to one or more groups of the search strategy in the title or abstract; articles in which obesity, bodyweight, or BMI was only a comorbidity occurring in a percentage of the patients and was not further correlated or analyzed as an immune aspect of COVID‐19; articles without an abstract; and articles from preprint servers. The results were then systematized following the immune system topics and synthesized accordingly.

## 3 RESULTS

**FIGURE 1:**
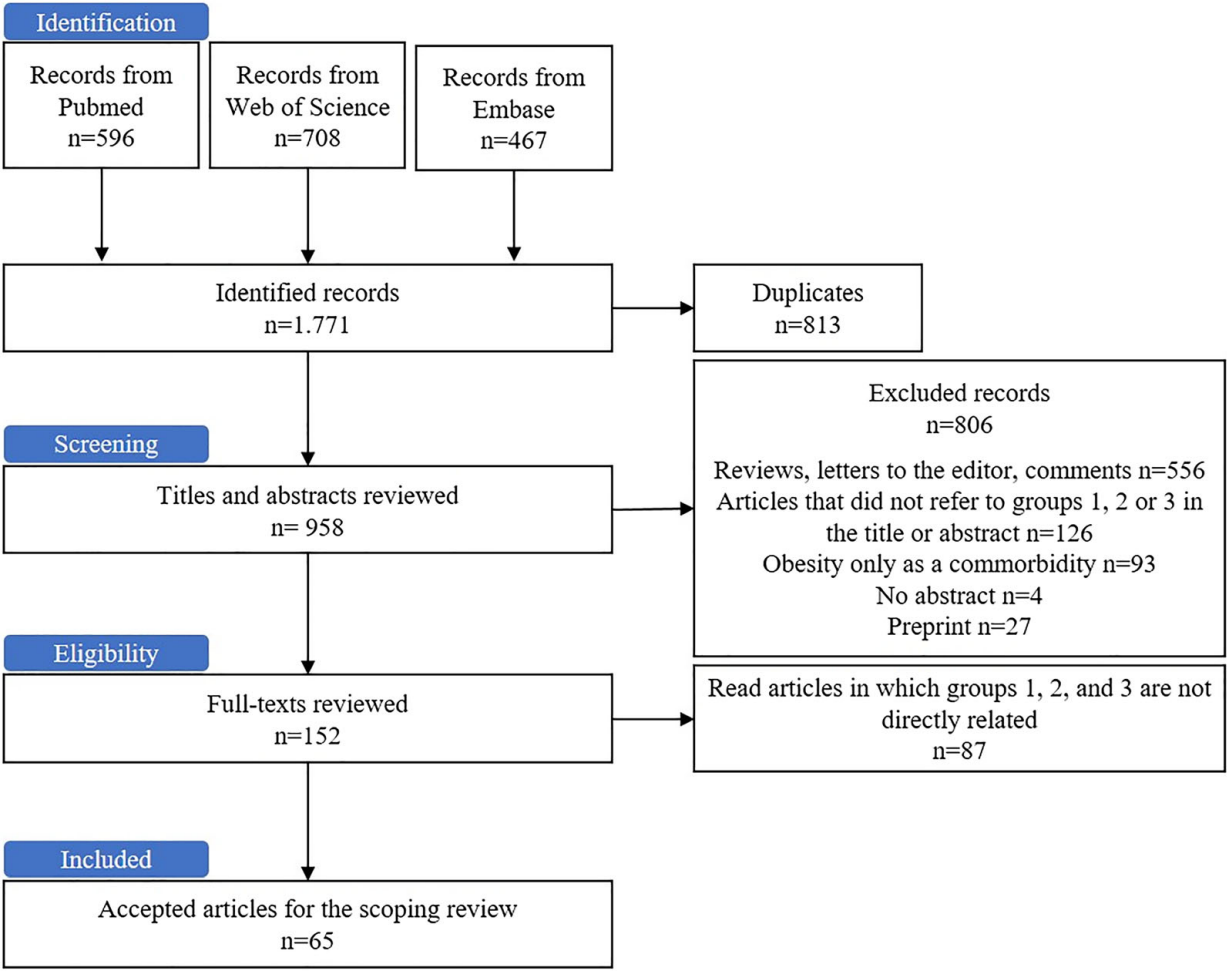
Flow chart of the literature search showing the screening process and results

A total of 525,735 articles referring COVID‐19 were published in the PubMed, Web of Science, and Embase databases. The search combining the three groups of keywords returned 1771 references: 596 from PubMed, 708 from Web of Science, and 467 from Embase. After filtering for duplicates from different databases, 917 references were further screened for eligibility. Out of these, 152 full texts were reviewed after excluding unrelated reviews, letters to the editor and comments (*n* = 556), articles that did not refer to one or more groups of keywords in their titles or abstracts (*n* = 127), articles in which obesity was described only as a comorbidity (*n* = 93), articles without an abstract (*n* = 4), and articles from preprint servers (*n* = 27). Eighty‐six articles were discarded because they failed to interrelate groups 1, 2, and 3 of the keywords. Finally, a total of 65 articles were included in this scoping review (Figure 1). During this period, two articles were published using an experimental model with obesity, four articles involving children, and 31 articles involving adults. Most of them evaluated hospitalized patients, many of whom were admitted to an intensive care unit, dead on arrival, or survivors without information on sex or race.

**FIGURE 2:**
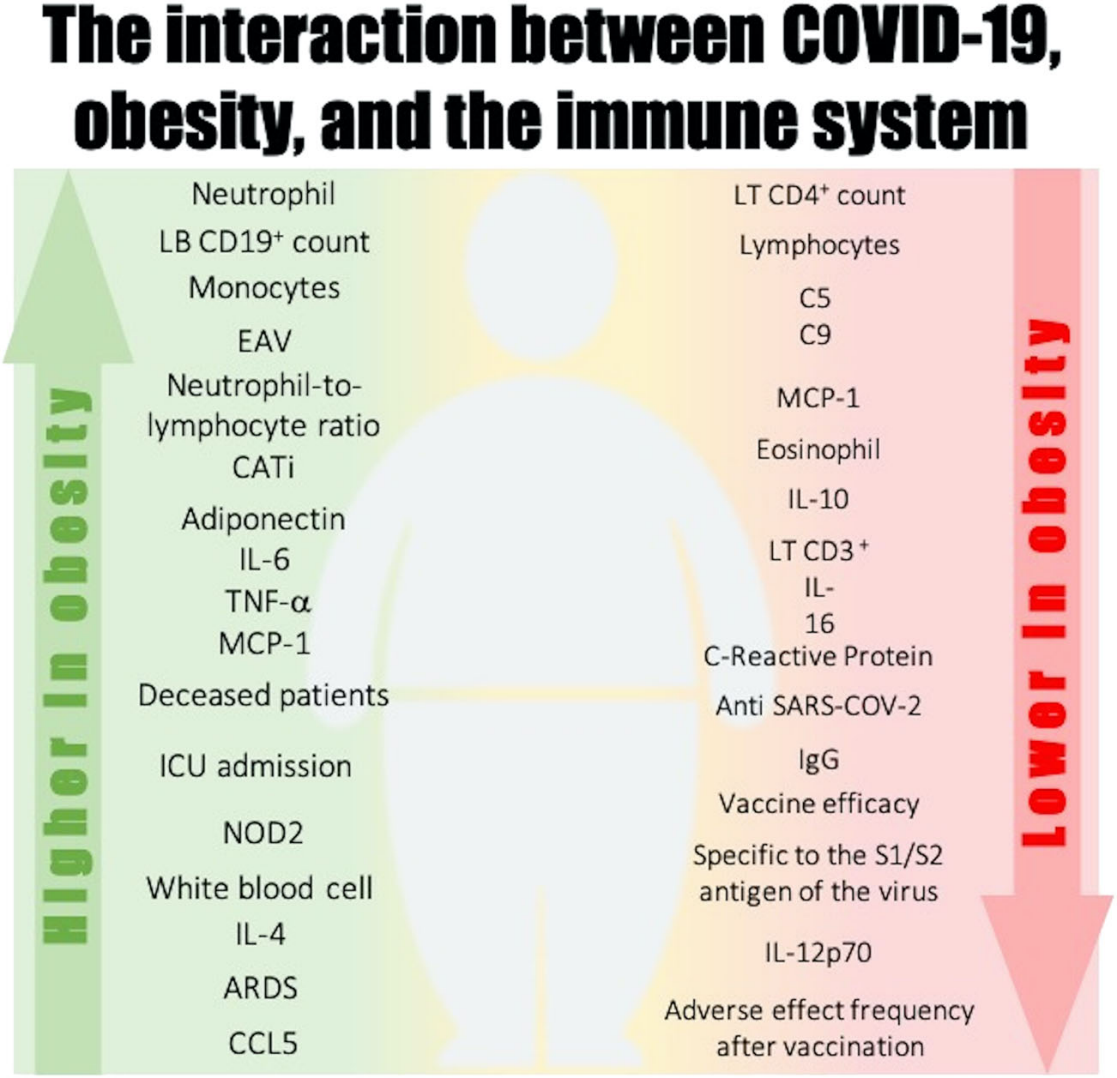
Main findings on the interaction between COVID‐19, obesity, and the immune system. Disease outcomes, pathways, cells, and molecules with higher levels or positively associated with obesity are shown in green, while disease outcomes, pathways, cells, and molecules with lower levels or negatively associated with obesity are shown in red. ARDS: acute respiratory distress syndrome; BMI: body mass index; CATi: cardiac adipose tissue; EAV: epicardial adipose tissue; ICU: intensive care unit; LT: T lymphocyte; T2DM: type 2 diabetes mellitus; MCP‐1: monocyte chemoattractant protein‐1

**FIGURE 3:**
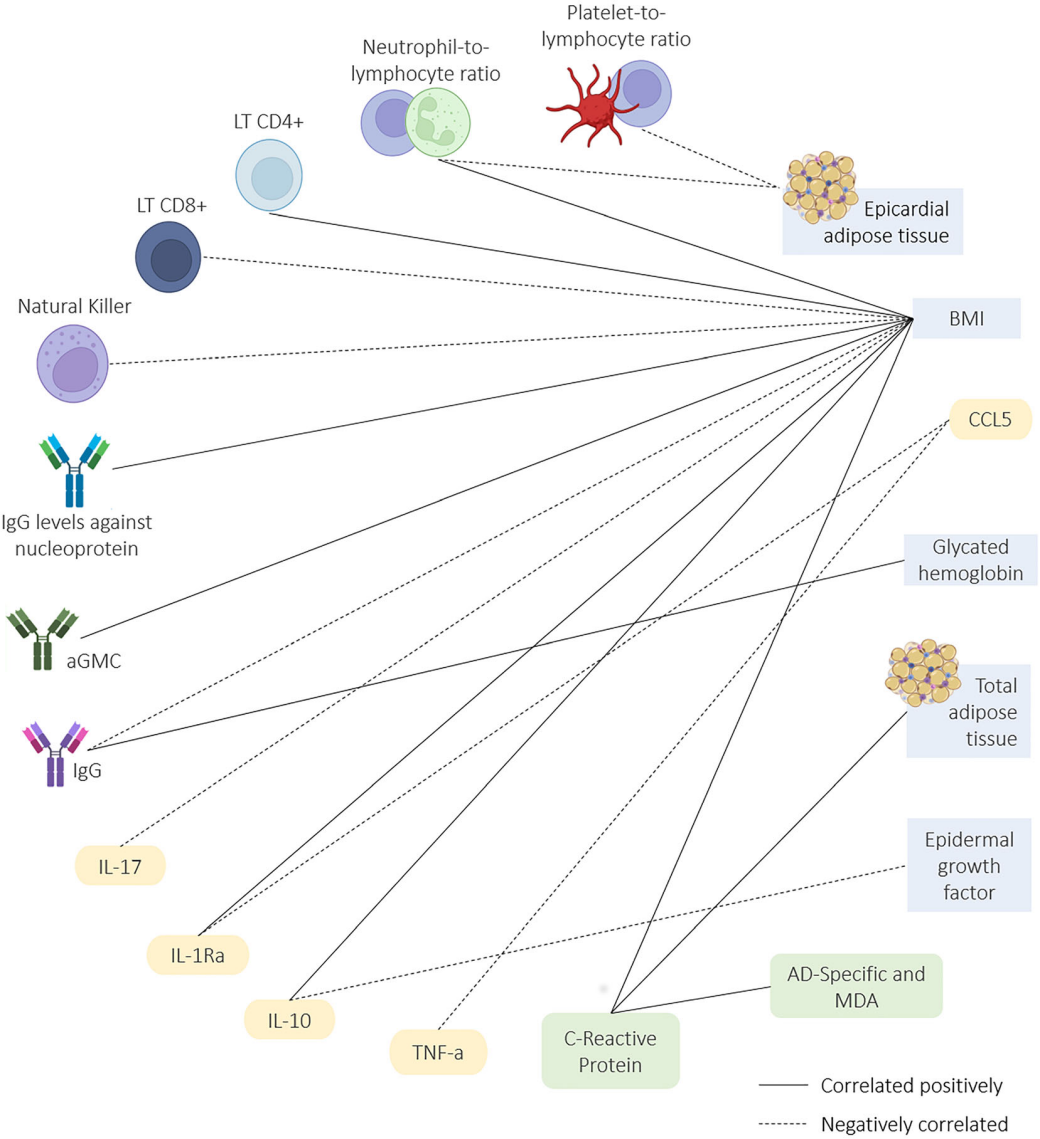
Correlations networks of immune response and the adipose tissue with COVID‐19. The continuous lines show positive correlations, and traced lines show negative correlations between immune cells and molecules and adiposity parameters or among themselves. The correlations were retrieved from the reviewed articles and are all statistically significant (*p* < 0.05) AD: adipocyte‐derived; BMI: body mass index; CCL: C‐C chemokine ligand; IL: interleukin; LT: T lymphocyte; TNF: tumor necrosis factor; MDA: malondialdehyde; aGMC: geometric mean antibody concentration

Several biomarkers have been proposed in an attempt to understand the impact of obesity on the severity of SARS‐COV‐2 infection (Figure 2). Individuals with underlying conditions that cause an immune‐compromised state, such as obesity, are considered vulnerable to this infection. The cells and molecules of the immune response are important determinants of viral infections, including coronaviruses, not only in the antiviral defense but also in the disease progression, severity, and clinical outcomes. SARS‐CoV‐2 infection can activate innate and adaptive immune systems and result in massive inflammatory responses later in the disease. Furthermore, these parameters are correlated with BMI status and adiposity (Figure 3).

### 3.1 Obesity, COVID‐19, and immune cells

A number of studies have reported that the hematological parameters in hospitalized COVID‐19 patients presented higher neutrophil counts, as well lower lymphocyte and eosinophil counts.^25^, ^26^, ^27^, ^28^, ^29^, ^30^, ^31^, ^32^, ^33^, ^34^ Cell count of the innate immunity was altered in infected patients with a higher BMI in several studies.^29^, ^33^, ^34^, ^35^, ^36^ The frequency of dendritic cells and monocytes was negatively correlated with BMI in aged COVID‐19 patients,^36^ while natural killer (NK) cells decreased with BMI in young COVID‐19 patients.^36^ In general, the neutrophil‐to‐lymphocyte ratio (NLR) is positively associated with adiposity and severity in COVID‐19‐positive patients.^25^, ^30^, ^31^ In contrast, the lymphocyte count significantly dropped in patients with obesity as compared to those without obesity, and this percentage was significantly associated with death in patients with obesity in univariate and multivariate analyses.^34^, ^37^ In addition, sedentary and children with obesity had a lower frequency of T regulatory cells associated with a higher frequency of Th1 cells.^38^ The frequency of CD4^+^ cells in the subgroup of COVID‐19 patients with obesity was significantly decreased in the severe group as compared to the non‐severe group.^33^ Moreover, the percentage of CD19^+^ cells was higher in overweight and patients with obesity as compared to normal‐weight patients.^33^ It was observed that the lymphocyte‐to‐C‐reactive protein (CRP) ratio (LCR) and the lymphocyte‐to‐platelet ratio (LPR) is lower as well.^25^ The lymphocyte and platelet counts are negatively correlated with BMI.^34^, ^39^ The phenotypic profile of innate and adaptative cells is summarized in Table 2 and Figures 2 and 3.

**TABLE 2:** Obesity, COVID‐19, and immune cells

Observed characteristic	Sample	Main findings	Reference
Neutrophil‐to‐lymphocyte ratio (NLR)	125 adult (older than 18 years of age) patients with medical records available from admission to discharge (death or survival).	NLR at day 1 was predictive of intubation, thus worse outcome. A cutoff value of 4.94 was established as optimal. BMI was not associated with NLR values above or below/equal to the cutoff.	30
	776 patients (median age 60.5 years, 61.4% women, 75% non‐Hispanic Black). A higher frequency of women had obesity than men (63.8% vs 41.6%). Women also had higher BMI than men.	More women had NLR higher than 6. NLR higher than 6 was significantly associated with Intensive Care Unit (ICU) admission in both men and women, while a ratio higher than 6 was associated with invasive mechanical ventilation and death in women but not in men.	31
	100 patients (mean age 55.5 years, 68% male) with COVID‐19.	NLR was positively correlated with epicardial adipose volume but was not correlated with epicardial adipose density.	
Leukocytes	244 patients diagnosed with COVID‐19 and cardiovascular disease (hypertension, coronary heart disease, or heart failure). The patients were categorized into critical (*n* = 36) and non‐critical (*n* = 208) groups according to China's National Health Commission. Critically ill patients had higher BMI than non‐critical patients at admission.	Neutrophil and monocyte counts were higher in critically ill patients at admission. Critically ill patients had lower lymphocyte count at admission than non‐critical patients.	29
	22 adult (median age: 58.5 years) COVID‐19 positive patients admitted to the ICU. The patients were divided into lean (*n* = 10) and patients with obesity (*n* = 12) using the percentage of fat mass and age.	There were no differences between the groups in the neutrophil count in blood at the baseline. Comparing the count at the baseline and 10 days after admission, there were no changes in the neutrophil between the groups (baseline vs 10 days). There were no differences between the groups in the lymphocyte count at the baseline. Comparing the count at the baseline and 10 days after admission, there was an increase in the count at day 10 in the lean group only.	26
	13 deceased young (14–40 years of age) and 40 young age‐ and sex‐matched survivors from COVID‐19.	Deceased patients had higher BMI than survivors and presented lower eosinophil and lymphocyte counts.	32
	95 Chinese patients with positive PCR test divided into patients with obesity (*n* = 36, BMI ≥ 25 kg/m^2^) and without obesity (*n* = 59, BMI < 25 kg/m^2^) groups.	Monocyte and lymphocyte counts were higher in patients with obesity than individuals without obesity.	28
	24 intubated patients treated in the ICU for Acute Respiratory Distress Syndrome (ARDS) of varying degrees of severity and 26 patients who were breathing spontaneously without ARDS. The median age of the patients was 65. The patients that developed ARDS more frequently had a pre‐existing respiratory disease (58% vs 42%) and were more frequently patients with obesity (46% vs 23%) or overweight (38% versus 19%) as compared to those without ARDS.	The ARDS group had higher white blood cell counts than patients without ARDS on admission. White blood cell counts remained elevated for the entire observation period (9 days).	27
	463 COVID‐19 adult patients divided according to BMI in normal weight (18.5–23.9 kg/m^2^, *n* = 242), overweight (24–27.9 kg/m^2^, *n* = 179), and patients with obesity (>28 kg/m^2^, *n* = 42).	White blood cell count, neutrophil count, and basophil count were higher in overweight and patients with obesity as compared to normal weight. Neutrophil percentage, lymphocyte percentage, and platelet count were higher in overweight patients as compared to normal weight.	
	65 adult patients admitted to a hospital with SARS‐CoV‐2 infection detected by RT‐PCR. The patients were divided according to the length of stay in non‐prolonged length of stay (NPLOS, <26 days) and prolonged length of stay (PLOS, ≥26 days). PLOS group had a higher proportion of patients with obesity as compared to the NPLOS group.	PLOS patients had a higher neutrophil count than NPLOS patients. No difference between PLOS and NPLOS patients was observed regarding white blood cells, monocyte, lymphocyte, and platelet count. The Systemic Inflammatory Response Index ([neutrophils × platelets]/lymphocytes) and the Aggregate Index of Systemic Inflammation ([neutrophils × monocytes × platelets]/lymphocytes) were higher in PLOS than NPLOS patients. PLOS patients had higher NLR than NPLOS patients. NLR was positively correlated with hospital length of stay.	34
	50 pre‐pubertal children were divided based on their mean daily step count in sedentary (*n* = 17) and active (*n* = 33) groups.	Active children were younger and had lower BMI and fat percentages than sedentary children. Sedentary children had a lower frequency of Treg cells. Sedentary and children with obesity facing COVID‐19 quarantine period may have a pro‐inflammatory background.	38
	791 patients aged ≥18 years with 460 (58.2%) male and 363 (45.9%) with obesity.	The patients with obesity had lower absolute lymphocyte count (ALC) than individuals without obesity at days 1 and 2 post‐admission. The patients with obesity and diabetes had lower ALC at days 1 and 2 than patients without diabetes or diabetes only.	35
	A 31‐year‐old African American female with COVID‐19 positive diagnosis, morbid obesity, previous history of childhood asthma, and cutaneous psoriasis presented with 1 week of severe dyspnea on exertion, cough, fever, chills, and myalgia.	ALC within the normal range (20–43%) at admission and below the range on the last day before death.	40
	230 adult (age range: 20–52 years), home‐isolated COVID‐19‐positive patients divided into three groups according to their BMI: normal‐weight (*n* = 30, BMI < 25 kg/m^2^), overweight (*n* = 58, 25 < BMI < 30 kg/m^2^), and patients with obesity (*n* = 142, BMI ≥ 30 kg/m^2^). Blood samples were collected 5–7 days after the appearance of the symptoms.	The patients with obesity had a lower lymphocyte percentage than normal‐weight and overweight patients. Lymphocyte percentage was significantly associated with death in patients with obesity in univariate and multivariate analyses	37
	13 COVID‐19 adult patients under invasive mechanical ventilation who had received previous antiviral and/or anti‐inflammatory treatments (including steroids, lopinavir/ritonavir, hydroxychloroquine, and/or tocilizumab, among others) were treated with allogeneic adipose‐tissue derived mesenchymal stromal cells (AT‐MSC).	Out of six patients in which lymphocyte counts were measured by flow cytometry, an increase in the levels of total lymphocytes was observed in five of them (86%), as well as an increase in B (67%) and CD4+ and CD8+ (100%) T lymphocytes.	41
	96 patients hospitalized with SARS‐CoV‐2 infection.	Lymphocyte count (*r* = −0.23, *p* = 0.027) and platelet count (*r* = −0.44, *p* < 0.001) were negatively correlated with BMI.	39
	39 young (age <60 years) and 48 aged (age≥60 years) COVID‐19‐positive patients classified according to BMI into lean (BMI ≤ 24.9 kg/m^2^), overweight (25–29.9 kg/m^2^), and patients with obesity (≥30 kg/m^2^).	The frequency of proliferating CD4+ central memory T cells correlated positively (*r* = 0.3158, *p* = 0.0132) with BMI in aged patients, while a negative correlation (*r* = −0.6182, *p* = 0.0478) was observed of CD8+ cells secreting IFN‐γ with BMI in young patients. Additionally, the expression level of CD16 on NK lymphocytes was negatively correlated (*r* = −0.3538, *p* = 0.0060) with BMI in young patients only. The frequency of CD14 + CXCR6 + monocytes correlated positively with BMI (Spearman *r* = 0.3027, *p* = 0.0261) in young patients. There were no additional correlations in any of the groups regarding the frequency of total monocytes or monocyte subsets and BMI. Considering the total sample, the frequency of total DC (*r* = −0.3122, *p* = 0.012) and myeloid DCs (*r* = −0.2954, *p* = 0.0169) was significantly negatively correlated with BMI, while that of plasmacytoid DCs showed no correlation with BMI.	36
	126 patients older than 18 years old and with available SARS‐CoV‐2 RNA detection data. According to the duration of viral shedding, the patients were categorized as prolonged (≥28 days) or non‐prolonged (<28 days) shedders.	The patients with prolonged shedding had a higher BMI and a higher proportion of patients with obesity. Levels of NK cells on admission were higher in prolonged as compared to non‐prolonged shedders. Among the factors associated with prolonged shedding in a multivariable logistic regression, BMI and NK cells were associated with higher odds ratio, while CD4+ cells were associated with a lower odds ratio of prolonged shedding.	42
	463 COVID‐19 adult patients divided according to BMI in normal weight (18.5–23.9 kg/m^2^, *n* = 242), overweight (24–27.9 kg/m^2^, *n* = 179), and patients with obesity (>28 kg/m^2^, *n* = 42).	CD19+ cell count and percentage were increased in overweight and patients with obesity as compared to normal‐weight patients. CD3+ cell percentage was decreased in the blood of patients with obesity as compared to normal weight. Among the patients with obesity, CD4+ cell count was lower in severe patients as compared to non‐severe patients.	33
	600 patients with COVID‐19. The mean BMI of the cohort was 31.5 kg/m^2^, and 301 (50.2%) were classified as patients with obesity. Women comprised 45.4% (273) and had a higher percentage of obesity than men.	In multivariate analysis, absolute lymphocyte count and percentage of lymphocytes were positively associated with BMI after adjustment for age, sex, and race. Lower lymphocyte percent and higher ferritin and D‐dimer levels were significantly associated with ICU admission.	43
	A 9‐year‐old boy with obesity presenting fever, loss of appetite, and fatigability at admission. His mother had COVID‐19 one month before admission but the patient did not present any symptoms at the time. The case was treated as pediatric inflammatory multisystem syndrome (PIMS) associated with COVID‐19. The patient was treated with intravenous immunoglobulin in a dose of 0.5 g/kg/day for 5 days and methylprednisolone 2 mg/kg/day for 7 days.	The patient presented lymphopenia at the onset (1420/μL) but normalized levels at discharge (7 days later).	44
	C57BL/6 mice fed a normal diet or a high‐fat diet from 8 weeks of age to 12 weeks of age or 48 weeks of age.	At 48 weeks of age, the high‐fat diet‐fed mice had lower *Ace2* mRNA expression in their intestinal macrophages as compared to intestinal macrophages of normal diet‐fed mice.	45
	10 patients whose death was due to COVID‐19 infection (COVID‐19 group) from March to September 2020; 10 patients who had a premortem diagnosis of hypertension, type 2 diabetes mellitus, and chronic kidney disease and had died and had an autopsy performed during the same period (Control group); 5 patients with myocarditis whose autopsy occurred from 2015 to 2020 (Myocarditis group).	In some hearts of the COVID‐19 group (the article does not specify the *n*), there were a significant number of CD68+ cells seen in the epicardial adipose tissue.	46
Lymphocyte‐to‐CRP ratio (LCR)	100 patients (mean age 55.5 years, 68% male) with COVID‐19.	LCR negatively correlated with epicardial adipose tissue volume but not with epicardial adipose tissue density.	25
Platelet‐to‐lymphocyte ratio (PLR)	100 patients (mean age 55.5 years, 68% male) with COVID‐19.	PLR positively correlated with epicardial adipose tissue volume but not with epicardial adipose tissue density.	25

### 3.2 Obesity, COVID‐19, and immunological molecules

The proinflammatory cytokines and chemokines, such as IL‐6, IL‐8, tumour necrosis factor (TNF), IL‐17, IL‐12, IL‐1, IP‐10, MCP‐1, and CCL5, were generally increased with higher BMI.^27^, ^28^, ^36^, ^40^, ^47^, ^48^, ^49^, ^50^, ^51^, ^52^, ^53^, ^54^, ^55^, ^56^ Furthermore, high levels of IL‐10, an anti‐inflammatory cytokine, appear to be a hallmark of hyperinflammation during severe SARS‐CoV‐2 infection,^57^, ^58^ and some studies included in this review observed that IL‐10 levels predict poor outcomes in COVID‐19 patients with obesity.^32^, ^51^ In an attempt to reduce the high COVID‐19 mortality rates due to a cytokine storm, intensive research is underway examining the use of anti‐inflammatory drugs. Recent studies have supported the use of the IL‐6R inhibitor tocilizumab,^40^ and the use of colchicine in COVID‐19^47^ was associated with the reduction of multiple inflammatory mediators, including CRP, IL‐6, resistin, and vascular proteins in obesity. Visceral fat tissue is known for its proinflammatory effects, which are caused not only by the Angiotensin II system^59^ but also by adipokines, such as leptin.^60^, ^61^ Higher leptin levels have been demonstrated in SARS‐CoV‐2 ventilated patients and positively associated with high BMI.^62^ During these past 2 years, several other inflammation biomarkers, including CRP,^26^, ^27^, ^28^, ^29^, ^31^, ^32^, ^35^, ^37^, ^40^, ^41^, ^44^, ^63^ innate receptors,^52^, ^64^ complement,^47^, ^65^ and lipidic mediators^47^ have been associated with COVID‐19 severity in patients with obesity (Table 3, Figures 2 and 3).

**TABLE 3:** Obesity, COVID‐19, and immunological molecules

Observed characteristic	Sample	Main findings	Reference
Cytokines/adipokines	A 31‐year‐old African American female with COVID‐19 positive diagnosis, morbid obesity, previous history of childhood asthma, and cutaneous psoriasis presented with 1 week of severe dyspnea on exertion, cough, fever, chills, and myalgia.	Serum IL‐6 levels were 4.9 times higher than the upper limit of the reference range (0.0–15.5 pg/mL).	40
	Male Wistar rats were randomly divided into three experimental groups: control diet (Control, *n* = 6), high sugar‐fat diet (HSF, *n* = 6), or high sugar‐fat diet + γ‐oryzanol (HSF + γOz, *n* = 6) during 30 weeks.	Leptin levels were increased in the HSF group as compared to the control group and decreased in the HSF + γOz as compared to the HSF group. Adiponectin levels were increased in both HSF and HSF + γOz. Epididymal adipose tissue IL‐6, TNF‐α, and MCP‐1 were increased in the HSF group as compared to the control and HSF + γOz. PPAR‐γ expression in the adipose tissue was decreased in the HSF group and recovered in the HSF + γOz group. Authors advocate for the use of γOz as a natural supplement in COVID‐19.	48
	31 COVID‐19 positive patients requiring mechanical ventilation and 8 critically ill patients without COVID‐19.	COVID‐19 patients had higher BMI and higher serum leptin than non‐COVID‐19 patients. Leptin levels correlate with BMI only in COVID‐19 patients. The authors propose that leptin is increased due to ACE2‐angiotensin II disruption due to the viral infection. In this context, inflammation is exacerbated in the lungs because of higher concentrations of leptin and angiotensin II.	62
	Expression data of SARS‐CoV‐2‐infected and non‐infected human epithelial cells (Gene Expression Omnibus accession number GSE147507).	Using Pathvisio for visualizing the expression of genes of the leptin pathway, it was observed that *SOCS3, STAT1*, *NFKB1*, and *IL1B* were upregulated in infected cells.	66
	Expression data of 3 T3‐L1 adipose cells treated with TNF‐α (GSE87853).	TNF‐α treated cells do not change their expression of the GRP78 receptor, which this study shows as a molecule capable of binding to the spike protein of SARS‐CoV‐2 and may concentrate and accumulate the viral particles in ACE‐2 expressing cells.	67
	13 deceased young (14–40 years old) and 40 young age‐ and sex‐matched survivors.	Deceased patients had higher BMI than survivors and presented higher levels of IL‐10 and TNF‐α. No alterations were observed in the levels of IL‐6, IL‐8, IL‐1β, and IL‐2R.	32
	95 Chinese patients with positive PCR test divided into patients with obesity (*n* = 36, BMI ≥ 25 kg/m^2^) and without obesity (*n* = 59, BMI < 25 kg/m^2^) groups.	IL‐6 and IL‐4 levels were increased in the serum of patients with obesity, while IL‐10 showed no difference between the groups.	28
	35 patients with obesity and with metabolic syndrome adult patients were randomly assigned to the placebo (*n* = 18) and colchicine (*n* = 17) groups.	IL‐6, IL‐16, and resistin levels were significantly decreased in patients receiving colchicine, suggesting this drug as a potential therapy for COVID‐19.	47
	24 intubated patients treated in the ICU for ARDS of varying degrees of severity and 26 patients who were breathing spontaneously without ARDS. The median age of the patients was 65 (IQR 58–76). The patients that developed ARDS more frequently had a pre‐existing respiratory disease (58% versus 42%) and were more frequently patients with obesity (46% vs 23%) or overweight (38% vs 19%) as compared to those without ARDS.	The ARDS group had higher IL‐6 levels on admission as compared to patients without ARDS. The ARDS group exhibited persistently elevated IL‐6 levels over the observation period of 6 days.	27
	67 COVID‐positive patients admitted to the ICU and divided into patients with obesity (BMI ≥ 30 kg/m^2^, *n* = 18, 72% class I obesity, 28% class II obesity) and without obesity (BMI < 30 kg/m^2^, *n* = 49) groups.	Pro‐inflammatory cytokines were highest at ICU admission and decreased over time, both in the patients with obesity and group without obesity (*p* < 0.05 for all cytokines). Except for slightly higher IP‐10 levels in patients with obesity versus individuals without obesity at days 9–10. BMI did not correlate with concentrations of IL‐6, TNF‐α, and IP‐10 at ICU admission (*r* = −0.09, *p* = 0.61, *r* = 0.03, *p* = 0.99 and *r* = 0.28, *p* = 0.11, respectively) or any other cytokine measured (*r* values ranging from −0.11 to 0.06; *p* values all >0.50).	50
	781 adult patients who were hospitalized due to COVID‐19. 349 patients were patients with obesity (BMI ≥ 30 kg/m^2^).	Initial and peak IL‐6 levels were not different between patients with obesity and individuals without obesity. Peak D‐dimer levels were higher in patients with obesity as compared to those without obesity. D‐dimer levels were associated with ICU admission, hypoxemic respiratory failure, intubation, vasopressor use, and death.	68
	Young (2–6 months old) and old (20–24 months old) C57BL/6 mice infected with the murine coronavirus MHV‐A59, whose infection is similar to SARS‐CoV‐2. In some experiments, mice were fed a normal diet or a ketogenic diet.	Compared to infected young mice, infected old mice had higher expression of *Il1b*, *Tnf*, and *Il6* genes in the visceral adipose tissue. Infected animals had higher *Casp1* expression in the visceral adipose tissue and higher inflammasome activation. Old mice under a ketogenic diet had lower expression of *Il1b*, *Tnf*, *Il6*, *Nlrp3*, and *Casp1* and lower Procaspase 1 activation.	55
	39 young (age <60 years) and 48 aged (age≥60 years) COVID‐19‐positive patients classified according to BMI into lean (BMI ≤ 24.9 kg/m^2^), overweight (25–29.9 kg/m^2^), and patients with obesity (≥30 kg/m^2^).	CCL5 levels were higher in aged overweight and patients with obesity than normal‐weight patients, while MCP‐1 was lower in patients with obesity aged as compared to lean aged patients and lower in overweight young patients as compared to lean young patients. IL‐10 levels correlated negatively and EGF positively with BMI in young patients. TNF‐α and IL‐1RA correlated negatively and CCL5 correlated positively with BMI in aged patients.	36
	4925 adults (≥18 years old) with laboratory‐confirmed COVID‐19 who were hospitalized across the United States. According to BMI, patients were divided into six categories: <18.5, 18.5–24.9, 25–29.9, 30–34.9, 35–39.9, and >40 kg/m^2^.	In a multivariable‐adjusted regression, there was no relationship between BMI and IL‐6, CRP, or D‐dimer levels. Results did not change if BMI was treated as a continuous variable or a categorical one.	69
	3530 adult COVID‐19 patients divided according to BMI into 6 categories: underweight, normal weight, overweight, class I obesity, class II obesity, and class III obesity.	No association was noted between BMI categories and IL‐6 levels. BMI and IL‐6 were independently associated with in‐hospital mortality, intubation, and severe pneumonia.	70
	81 patients with type‐II diabetes and COVID‐19 (positive SARS‐CoV‐2 PCR).	Increased cardiac adipose tissue (CATi) was associated with early mortality, both as a continuous variable and as a dichotomous variable. CATi and IL‐6 were significantly increased in the ICU patients.	53
	406 individuals from the UK without COVID‐19 and with data of 35 inflammatory molecules. Genotypic data from these individuals were collected and compared with genotypic data from severe COVID‐19 cases to determine the genetic susceptibility to severe infection.	In linear regression, the genetic risk for severe COVID‐19 interacted with BMI to negatively affect IL‐17 levels.	54
	167 hypertensive patients with COVID‐19. The mean age was 54.1 ± 12.3 years, 57 (34.1%) female, 88 (52.7%) were patients with obesity, 41 (24.6%) had diabetes, and 29 (17.4%) had dyslipidemia.	In patients with obesity and diabetes, the likelihood of COVID‐19 progression to more severe forms of COVID‐19 increased from 0.1% with low levels of IL‐10 and IL‐12 (p70) to >80% with levels of these cytokines exceeding the sensitivity thresholds of 90%. In addition, the risk of progression to severe disease in the presence of three clinical comorbidities was 1.0% with levels of IL‐10 and IL‐12 (p70) below thresholds and 97.5% with levels above thresholds.	51
	60 COVID‐19 patients with mild (not hospitalized, *n* = 11), moderate (hospitalized but not requiring intensive care, *n* = 25), and severe (admission to the intensive care unit [ICU] or death, *n* = 24) disease. 25.0% had a normal weight, 43.3% were overweight, 31.7% had obesity, and 20% had diabetes mellitus.	The patients with moderate disease had higher adiponectin to leptin ratio than mild patients. The ratio was positively correlated with CRP levels (*r* = 0.293, *p* = 0.023). The highest tertile presented a higher frequency of patients being admitted to the ICU than the first tertile.	71
	70 patients ‐ 43 women and 27 men, median age 58.5 (49.0–67.0) years with COVID‐19 proven by RT‐PCR and 20 uninfected controls were included in the study.	Serum levels of chemerin and omentin were decreased in COVID‐19 in comparison to control patients. The comparison of COVID‐19 patients with different insulin sensitivity and control groups demonstrated that both patients with HOMA‐IR ≤ 3 or > 3 had significantly lower chemerin levels in comparison to the control group. Omentin was decreased only when comparing HOMA‐IR ≤ 3 COVID‐19 subgroup with healthy volunteers.	72
	11 adult patients with obesity received resveratrol or placebo for 30 days.	Resveratrol supplementation decreased the expression of the leptin gene and the SARS‐CoV‐2 receptor ACE2 in the adipose tissue.	73
	Data from 18 deceased patients whose primary cause of death was respiratory failure, 14 due to COVID‐19 (GSE151764). Among the 14 patients with COVID‐19, 8 had hypertension (group 1), 3 hypertension + type 2 diabetes mellitus (group 2), and 3 hypertension + type 2 diabetes mellitus + obesity (group 3).	15 genes were induced only in patients of Group 3 relative to controls. These genes included those associated with leptin (*LCK*, *IRF1*, *VCAM1*, *SNAI1*), MHC‐class I and II (*HLA‐A*, *HLA‐C*, *HLA‐DQA2*), and immune regulatory functions (*IL10*, *CD40*, *NT5E*, *CORO1A*). Considering only the 14 COVID‐19 patients, 17 genes were exclusively associated with BMI; among them those associated with cytokine signaling and immune response (*CD40LG*, *CXCR2*, *ADORA2A*, *PIK3CD*, and *CD37*) and receptors for the Fc region of IgG molecules (*FCGR3A* and *FCGR3B*) were downregulated.	74
	Qiagen Knowledge Base (QKB) and Ingenuity Pathway Analysis (IPA) tools for exploring potential pathways affected by the palmitic acid (PA) – a free‐fatty acid commonly enriched in high‐fat diets and elevated in the circulation of patients with obesity.	35 molecules overlapped PA‐ and COVID‐19‐enriched molecules, including COX‐2, IL‐1β, IFN‐β1, IL‐6, CCL2, CXCL8, and CCL5. Examining the paths by which PA might affect the coronavirus pathogenesis pathway, 38 shortest pathways were found. Among the molecules in these pathways, many of them were immune related, including FOS, EEF1A1, IL‐1β, IFN‐β1, IL‐6, CCL2, CXCL8, CCL5, and PTGS2. Contrary to the proinflammatory activity of PA, the unsaturated n‐3 fatty acids attenuate the activity of the inflammatory mediators and may be protective in the infection.	49
C‐reactive protein (CRP)	776 patients (median age 60.5 years, 61.4% women, 75% non‐Hispanic Black). A higher frequency of women had obesity than men (63.8% vs 41.6%). Women also had higher BMI than men.	CRP levels higher than 41.2 mg/dL were associated with ICU admission, invasive mechanical ventilation, and death in both men and women.	31
	244 patients diagnosed with COVID‐19 and cardiovascular disease (hypertension, coronary heart disease, or heart failure). The patients were categorized into critical (*n* = 36) and noncritical (*n* = 208) groups according to the interim guidance of China's National Health Commission.	Critically ill patients had higher CRP levels at admission than non‐critical patients.	29
	791 patients aged ≥18 years with 460 (58.2%) male and 363 (45.9%) with obesity.	CRP levels at day 1 or 2 did not differ between patients with obesity or individuals without obesity. The patients with obesity and diabetes had higher levels of CRP than the patients without these conditions.	35
	A 31‐year‐old African American female with COVID‐19 positive diagnosis, morbid obesity, previous history of childhood asthma, and cutaneous psoriasis presented with 1 week of severe dyspnea on exertion, cough, fever, chills, and myalgia.	CRP levels at admission were approximately 16 times above the reference range (<0.50 mg/dL).	40
	22 adult COVID‐19 positive patients (median age: 58.5 years) admitted to the ICU divided into lean (*n* = 10) and patients with obesity (*n* = 12) groups using the percentage of fat mass and age.	There were no differences between the groups in CRP levels at the baseline. Comparing CRP levels at the baseline and 10 days after admission, there was a significant decrease in the dosage at day 10 in the lean group only.	26
	13 COVID‐19 adult patients under invasive mechanical ventilation who had received previous antiviral and/or anti‐inflammatory treatments (including steroids, lopinavir/ritonavir, hydroxychloroquine, and/or tocilizumab, among others) were treated with allogeneic adipose‐tissue derived mesenchymal stromal cells (AT‐MSC).	There was a decrease in CRP levels in 8 out of 9 patients that improved clinically 5 days after AT‐MSC therapy.	41
	230‐adult (age range: 20–52 years), home‐isolated COVID‐19 positive patients divided into three groups according to their BMI: normal‐weight (*n* = 30, BMI < 25 kg/m^2^), overweight (*n* = 58, 25 < BMI < 30 kg/m^2^), and patients with obesity (*n* = 142, BMI ≥ 25 kg/m^2^). Blood samples were collected 5–7 days after the appearance of symptoms.	CRP levels were significantly elevated in patients with obesity as compared to normal‐weight and overweight patients. CRP levels were significantly associated with death in patients with obesity in both univariate and multivariate analyses.	37
	13 deceased (dead) young (14–40 years old) and 40 young age‐ and sex‐matched survivors from COVID‐19.	Deceased patients had higher BMI than survivors and presented higher levels of CRP.	32
	95 Chinese patients with positive PCR test divided into patients with obesity (*n* = 36, BMI ≥ 25 kg/m^2^) and without obesity (*n* = 59, BMI < 25 kg/m^2^) groups.	CRP levels were increased in patients with obesity as compared to individuals without obesity.	28
	35 patients with obesity and with metabolic syndrome adult patients were randomly assigned to the placebo (*n* = 18) and colchicine (*n* = 17) groups.	CRP levels were decreased following colchicine treatment. The authors suggest colchicine as a possible treatment in COVID‐19.	47
	150 patients (64.7% male, mean age 64 ± 16 years), divided into intubated (*n* = 35) and non‐intubated (115) groups based on their severity. Intubated patients had higher visceral adipose tissue (VAT) than non‐intubated.	Intubated patients were older (*p* = 0.009) and had higher CRP levels (*p* = 0.003). CRP correlated positively with the area of total adipose tissue (TAT) and VAT. Increasing age, lymphocytes, CRP, LDH, D‐Dimer, LSS, total abdominal fat, and VAT were found to have a significant univariate association with the need for intensive care. Age‐ and gender‐adjusted CRP was significantly higher in patients with worse clinical outcomes.	75
	24 intubated patients treated in the ICU for respiratory distress syndrome ARDS of varying degrees of severity and 26 patients who were breathing spontaneously without ARDS. The median age of the patients was 65 (IQR 58–76). The patients that developed ARDS more frequently had a pre‐existing respiratory disease (58% vs 42%) and were more frequently patients with obesity (46% vs 23%) or overweight (38% vs 19%) as compared to those without ARDS.	The ARDS group had a higher CRP on admission as compared to patients without ARDS. The ARDS group exhibited persistently elevated CRP levels over the observation period of 9 days.	27
	2466 adults hospitalized with PCR‐confirmed SARS‐CoV‐2 infection.	Compared with overweight patients, patients with obesity had a higher risk for intubation or death, with the highest risk among those with class 3 obesity. BMI was not correlated with admission CRP levels.	76
	124 adult patients (age>18 years) divided into positive (*n* = 52) and negative (*n* = 72) cases according to a RT‐PCR test.	BMI correlated positively with CRP levels (*r* = 0.27, *p* = 0.05) in the positive group. Positive patients had higher BMI than negative patients.	63
	A 9‐year‐old boy with obesity presenting fever, loss of appetite, and fatigability at admission. His mother had COVID‐19 one month before admission but the patient did not present any symptoms at the time. The case was treated as pediatric inflammatory multisystem syndrome (PIMS) associated with COVID‐19. The patient was treated with intravenous immunoglobulin in a dose of 0.5 g/kg/day for 5 days and methylprednisolone 2 mg/kg/day for 7 days.	The patient presented high CRP at the onset (>160 mg/L) but normalized levels at discharge (7 days later).	44
	318 patients with COVID‐19 who had undergone computed tomography (CT) of the chest. Total, subcutaneous, visceral, and intermuscular adipose tissue were measured (TAT, SAT, VAT, and IMAT, respectively).	Increasing levels of VAT were associated with increased CRP levels in a multivariate linear regression model. TAT, VAT, and IMAT were associated with hospitalization and mechanical ventilation or death.	77
	30 patients with a positive PCR test (15 lean and 15 patients with obesity) and 30 age‐ and BMI‐matched patients with pre‐pandemic plasma samples. Anti‐SARS‐CoV‐2, neutralizing, autoimmune (directed against malondialdehyde (MDA), and adipocyte‐derived antigens (AD) antibody levels were measured by ELISA.	CRP levels were higher in infected patients with obesity than in infected lean patients. CRP levels were positively correlated with MDA‐ (*r* = 0.74, *p* < 0.0001) and AD‐specific (*r* = 0.44, *p* = 0.002) antibodies.	78
	781 adult patients who were hospitalized due to COVID‐19. 349 patients were patients with obesity (BMI ≥ 30 kg/m^2^).	Initial and peak CRP levels were higher in patients with obesity as compared to patients without obesity. CRP levels were associated with ICU admission, hypoxemic respiratory failure, death, intubation, and vasopressor use.	68
	90 patients with SARS‐CoV‐2‐related pneumonia. 64.4% males and median age of 61 years.	CRP showed a significant difference when it reached its maximum levels during hospitalization. Maximum CRP levels were 92 (interquartile range [IQR]: 48–122) mg/L in normal‐weight patients, 140 (IQR: 82–265) mg/L in overweight patients, and 117 (IQR: 67–160) mg/L in patients with obesity (*p* = 0.037).	79
	29 adult patients admitted to the ICU due to COVID‐19. The patients were treated with the IL‐6R inhibitor tocilizumab (8 mg/kg to a maximum of 800 mg) in a single dose 24 h after ICU admission. Models were developed to evaluate if bodyweight would be a factor for dosage.	CRP levels decreased when tocilizumab was administered. In the sample, bodyweight did not affect the clearance of tocilizumab. Moreover, a total dose of 600 mg was as effective as the 800 mg dose.	80
Innate receptors	10‐week‐old control rats (*n* = 5) and metabolic syndrome rats untreated (*n* = 5), treated with candersatan (*n* = 5, 10 mg/kg), or treated with captopril (*n* = 5, 50 mg/kg).	Treated rats presented increased expression of the anti‐inflammatory receptors angiotensin II receptor type 2 (AT2) and Mas receptor (MasR) and decreased expression of the proinflammatory receptor AT1. Human alveolar type II cells treated with these drugs and the Spyke protein of SARS‐CoV‐2 presented lower expression of inflammatory cytokines (TNF‐α, IL‐6, and CCL‐2) than untreated cells, exposed to the Spyke protein.	52
	Whole blood RNA‐seq data from non‐infected patients with obesity (*n* = 20) and without obesity (*n* = 21) individuals.	NOD2 expression was higher in patients with obesity as compared to individuals without obesity. Obesity and older age lead to higher expression of CD147‐related genes on immune cells.	64
Complement	35 patients with obesity and with metabolic syndrome adult patients randomly assigned to the placebo (*n* = 18) and colchicine (*n* = 17) groups.	C5a and C9 were decreased in patients receiving colchicine.	47
	Skin biopsy from the deltoid region of 14 adults (age range: 28 to 73 years old) COVID‐19‐positive patients with severe (*n* = 13) or moderate (*n* = 1) disease.	Immunohistochemical staining revealed that there was a significant deposition of C5b‐9, C3d, and C4d in the endothelium of the subcutaneous adipose tissue (SAT) and vascular damage. Moreover, colocalization of complement proteins and SARS‐CoV‐2 was observed in the SAT.	65
Lipid mediators	35 adult patients with obesity and metabolic syndrome were randomly assigned to the placebo (*n* = 18) and colchicine (*n* = 17) groups.	COX‐2 was downregulated in patients with obesity receiving colchicine.	47
Furin protein	166 children in the Pediatric Osteoporosis Prevention (POP) study collected at mean age (standard deviation [SD]) 9.9 (0.6) years.	The mean (95% confidence intervals ‐ CI) furin normalized protein expression (NPX) for children with obesity, overweight, and low‐to‐normal weight was 8.0 (IQR: 7.8–8.3), 7.5 (IQR: 7.4–7.6), and 7.3 (IQR: 7.3–7.4), respectively, corresponding to 62% higher furin levels in children with obesity and 15% higher in children with overweight. Regression analysis showed that furin levels were higher in overweight children and in children with obesity as compared to children with low‐to‐normal weight. Serum furin was statistically correlated with BMI (*r* = 0.39, *p* < 0.001), total body fat mass (*r* = 0.40, *p* < 0.001), trunk fat mass (*r* = 0.41, *p* < 0.001), body fat percentage (*r* = 0.42, *p* < 0.001), triglycerides (*r* = 0.35, *p* < 0.001), leptin (*r* = 0.45, *p* < 0.001), A‐FABP (*r* = 0.48, *p* < 0.001), hsCRP (*r* = 0.33, *p* < 0.001), and IL‐6 (*r* = 0.43, *p* < 0.001), but not IL‐8 (*r* = 0.09, *p* = 0.26).	81
Antibodies	2547 individuals (mainly medical staff, older than 18 years old) from metropolitan Detroit and New York areas with a previous positive test for SARS‐CoV‐2.	Of 2547 subjects, 160 were IgG negative. Analyzing positive and negative IgG patients under BMI categories (under/normal weight, overweight, obesity, severe obesity), it was observed that the proportion of IgG negative patients decreased with increased body weight, suggesting that patients with obesity are more likely to maintain circulating antibodies. In a multivariate model, under/normal weight status remained significantly associated with the absence of serum antibodies.	82
	124 adult patients (age>18 years) divided into positive (*n* = 52) and negative (*n* = 72) cases according to a RT‐PCR test.	BMI negatively correlated (*r* = −0.3, *p* < 0.05) with Spike‐specific IgG levels assessed by ELISA in the positive patients.	63
	4085 subjects who have had a health check‐up at 16 health centers in South Korea. Seroprevalence was determined through electrochemiluminescence immunoassay (ECLIA) by using the Elecsys Anti‐SARS‐CoV‐2 (Roche Elecsys, Mannheim, Germany) kit.	Seroprevalence of anti‐SARS‐CoV‐2 antibodies in the studied sample did not differ with BMI or any other biochemical parameters	83
	39 young (age <60 years) and 48 aged (age≥60 years) COVID‐19‐positive patients classified according to BMI into lean (BMI ≤ 24.9 kg/m^2^), overweight (25–29.9 kg/m^2^), and patients with obesity (≥30 kg/m^2^).	IgG levels against nucleoprotein positively correlated with BMI (*r* = 0.4315, *p* = 0.0108) in young patients only.	36
	424 adult (age≥18 years) patients positive for SARS‐CoV‐2 IgG antibodies with BMI data available.	The patients with obesity (BMI > 30 kg/m^2^) had higher anti‐SARS‐CoV‐2 levels than lean patients (BMI < 25 kg/m^2^). The patients with obesity with a non‐severe disease course presented higher levels of neutralizing antibodies as compared to their lean counterparts. BMI and HbA1c levels are positively correlated with IgG levels (*r* = 0.3694, *p* < 0.0001 for the correlation with BMI; *r* = 0.1993, *p* = 0.0159 for the correlation with HbA1c).	84
	643 adult participants with available blood samples. 363 participants had overweight/obesity (BMI > 25 kg/m^2^). IgG antibodies were detected by indirect chemiluminescence and seropositivity was defined by antibody levels ≥15 AU/mL.	In the multivariable regression, overweight/obesity showed a relationship with seropositivity for SARS‐CoV‐2. The presence of overweight/obesity and type 2 diabetes was significantly associated with seropositivity using unadjusted or adjusted models.	85
	12,314 patients with COVID‐19 positive serology and BMI data available. The patients were divided according to BMI into five categories: <18.5, 18.5–25, 25–30, 30–40, and ≥40 kg/m^2^. Serology was determined by ELISA. ELISA results were described as antibody titer for the analyses (<1:80, 1:80, 1:160, 1:320, 1:960, 1:2880), and a positive test was defined as antibody titers of 1:80 and above.	Positive serology increased with higher BMI. The highest titer occurred in a higher proportion of patients with obesity (BMI 30–40 and ≥40 kg/m^2^) than in the other BMI categories. This observation remained when patients were stratified by PCR result (positive or negative) and age (<50 years or >50 years). The categories of patients with BMI 25–30, 30–40, and ≥40 kg/m^2^ had a significant association with the highest titer in a univariate regression.	86
	30 patients with a positive PCR test (15 lean and 15 patients with obesity) and 30 age‐ and BMI‐matched patients with pre‐pandemic plasma samples. Anti‐SARS‐CoV‐2, neutralizing, autoimmune (directed against malondialdehyde (MDA), and adipocyte‐derived antigens (AD) antibody levels were measured by ELISA.	Infected patients with obesity had lower levels of anti‐SARS‐CoV‐2 and neutralizing antibodies than infected lean patients. Neutralizing antibodies were detected in a few patients with obesity. The levels of autoimmune antibodies were higher in infected patients with obesity as compared to infected lean and uninfected patients with obesity.	78

Additionally, it was found that patients with obesity have higher anti‐SARS‐CoV‐2 levels than lean patients^82^, ^84^, ^85^, ^86^ (Figures 2 and 3). However, other studies showed that seroprevalence against SARS‐CoV‐2 did not differ with BMI or any biochemical parameters.^78^, ^83^

### 3.3 Obesity, COVID‐19, and vaccines

High vaccine effectiveness rates for the total population were reported, with slightly lower rates for the elderly and those suffering from hypertension, diabetes, or obesity, although BNT162b2 and mRNA‐1273 were reported to have a high efficacy (>89%) in the population with obesity.^87^, ^88^ While vaccination does not eliminate the risk of hospitalization or death, the risks are significantly reduced,^89^ and this result did not differ between the patients with obesity and the general population in one study.^87^ After vaccination, the studies either showed no differences in antibody levels across BMI categories or slightly reduced antibody levels in patients with obesity.^89^, ^90^, ^91^, ^92^, ^93^, ^94^ Furthermore, the vaccines were expected to perform well in terms of side effects in the population with obesity, although some minor side effects had different occurrence rates depending on the BMI.^95^, ^96^ The vaccine efficacy, antibody response, and side effects after vaccination are summarized in Table 4.

**TABLE 4:** Obesity, COVID‐19, and vaccines

Observed characteristic	Sample	Main findings	Reference
Vaccine efficacy	Compilation of reports of clinical trials on the efficacy of various vaccines (some were not published as journal articles).	Pfizer‐BioNTech BNT162b2: In 13,218 individuals with obesity (BMI ≥ 30 kg/m^2^, age≥16 years), the vaccine efficacy was 95.4% (95% confidence interval [CI]: 86.0%–99.1%). When stratified by age, the efficacy in young adults with obesity (16–64 years of age) and older adults with obesity (≥65 years of age) was similar: 94.9% (95% CI: 84.4%–99.0%) and 100.0% (95% CI: 27.1%–100.0%), respectively. Moderna mRNA‐1273: Vaccine efficacy in individuals with severe obesity (BMI ≥ 40 kg/m^2^) was 91.2% (95% CI: 32.0%–98.9%). One case of severe disease was identified among 901 participants with severe obesity who were vaccinated, while 11 cases were identified among 884 participants with severe obesity who received a placebo. Post‐hoc analysis yielded an efficacy of 95.8% (95% CI: 82.6%–99.0%) in patients with obesity (BMI ≥ 30 kg/m^2^). Janssen/Johnson & Johnson Ad26.CoV2.S: In 12,492 participants with obesity (BMI ≥ 30 kg/m^2^), efficacy 14 days after the dose was 66.8% (95% CI: 54.1%–76.3%) and 28 days after the efficacy was 65.9% (95% CI: 47.8%–78.3%). No deaths occurred in the vaccine group, while six of seven deaths in the placebo group occurred among participants with obesity. AstraZeneca AZD‐1222: No data were provided in the primary safety and efficacy analysis.	88
	1,658,604 members of the Maccabi HealthCare Services, Israel. According to the vaccinal status, the patients were divided into only vaccinated (received two doses of the BNT162b2 vaccine) and only unvaccinated (did not receive any dose during the study).	Vaccine efficacy for infection in patients with obesity was lower than the general population (89.7%, CI: 88.6–90.7 vs 93%, CI: 92.6–93.4). Efficacy rates for mortality and hospitalization did not differ between the patients with obesity and the general population.	87
Antibody response after vaccination	248 health‐care workers from the Istituti Fisioterapici Ospitalieri (IFO), Rome, Italy who received the BNT162b2 vaccine and a booster dose 21 days after the first immunization. According to BMI, the studied sample was divided in underweight (*n* = 19), normal weight (*n* = 147), pre‐obesity (*n* = 56), and patients with obesity (*n* = 26).	After the booster dose, increasing BMI was associated with lower levels of antibodies specific to the S1/S2 antigen of the virus (*p* = 0.033). Using a multivariate linear regression accounting for potential confounding variables, BMI was not associated with the antibody levels after the vaccination.	93
	242 health‐care workers from the Istituti Fisioterapici Ospitalieri (IFO), Rome, Italy who received the first dose of the BNT162b2 vaccine.	A strong correlation (*p* = 0.001) was detected between geometric mean antibody concentration (aGMC) and BMI, with higher antibody levels in underweight versus pre‐obesity group (*p* = 0.026) and in the normal weight group versus pre‐obesity (*p* = 0.007). This association remained significant after adjusting for age and sex.	94
	256 adult participants from Kuwait who were vaccinated with the second dose of the BNT162b2 vaccine. According to BMI, the participants were divided in normal weight (*n* = 65), overweight (*n* = 117), and patients with obesity (*n* = 74). Blood samples were drawn at least 3 weeks after the vaccination.	Total IgG levels against SARS‐CoV‐2 assessed by ELISA did not vary across BMI categories. Neutralizing antibodies did not vary either. No relationship between obesity status and antibody levels was found in a regression analysis.	90
	562 employees of a hospital located in the German province of Schleswig‐Holstein who had taken the BNT162b2, Vaxzevria, or no vaccine. The antibody testing was performed using semiquantitative ELISA detecting IgG antibodies against the S1 domain of the SARS‐CoV‐2 spike protein.	There was no difference in IgG levels between patients with obesity and without obesity employees who had been vaccinated with BNT162b2 or Vaxzevria.	91
	447 health‐care workers of the Hanyang University Hospital, Korea who received both the first and second doses of the ChAdOx nCoV‐19 vaccine. Anti‐RBD antibody levels were assessed by immunoassay 4 weeks after the second dose. The subjects were divided according to BMI into four categories: <18.5, 18.5–22.9, 23.0–24.9, and ≥25.0 kg/m^2^.	No difference in antibody levels was found across the BMI categories. No association was found between BMI and antibody levels in univariate and multivariate analyses.	92
Infection risk after vaccination	1,240,009 adult (age≥18 years) users of the COVID‐19 Symptom Study mobile app, UK. Of these, a proportion of the users were divided into those with a positive RT‐PCR test or lateral flow antigen test (LFAT) 14 days after their first dose (case 1, *n* = 6030), a positive test 7 days after their second dose (case 2, *n* = 2370), a negative RT‐PCR or LFAT 14 days after their first dose but before the second (control 1, *n* = 6030), and a negative test 7 days after their second dose (control 2, *n* = 2370).	The frequency of no patients with obesity was significantly lower in total case 1 and aged case 1 (age≥60 years) than total control 1 and aged control 1 users. Both in univariate and multivariate analyses, no obesity is associated with lower odds ratio of a positive test after the first dose than obesity.	89
Adverse effect frequency after vaccination	627,383 users of the COVID‐19 Symptom Study mobile app, UK, who received the BNT162b2 vaccine (first dose: 282,103; second dose: 28,207) or the ChAdOx nCoV‐19 vaccine (first dose: 345,280).	After dividing the users into without obesity (BMI < 30 kg/m^2^) and patients with obesity (BMI ≥ 30 kg/m^2^), it was verified that a higher proportion of users with obesity had systemic effects after each dose of the BNT162b2 vaccine and local effects after the second dose of the same vaccine. It was also verified that a lower proportion of users with obesity had systemic effects after the dose of the ChAdOx nCoV‐19 vaccine, but with a higher proportion of users with obesity reporting local effects.	96
	1189 Spanish adults who received at least one dose of AstraZeneca/Vaxzevria, Pfizer, Moderna, or Janssen vaccines. Respondents were surveyed through Google Forms for adverse effect frequency after vaccination and classified according to BMI as underweight, normal weight, overweight, or patients with obesity (< 18.50, 18.50–24.99, 25.00–29.90, ≥ 30.00 kg/m^2^).	The frequencies of fever <38 °C, fever ≥38 °C, myalgia, arm soreness, redness, swelling, nausea, red, itchy, swollen or painful rash, headache, loss of appetite, sweating, chills, tiredness, sleepness, and dizziness were all lower in patients with obesity and higher in underweight and normal‐weight respondents after the first dose. After the second dose, this association remained for fever <38°C, fever ≥38°C, myalgia, arm soreness, redness, swelling, headache, loss of appetite, sweating, chills, tiredness, sleepness, and dizziness.	95

## 4 DISCUSSION

**FIGURE 4:**
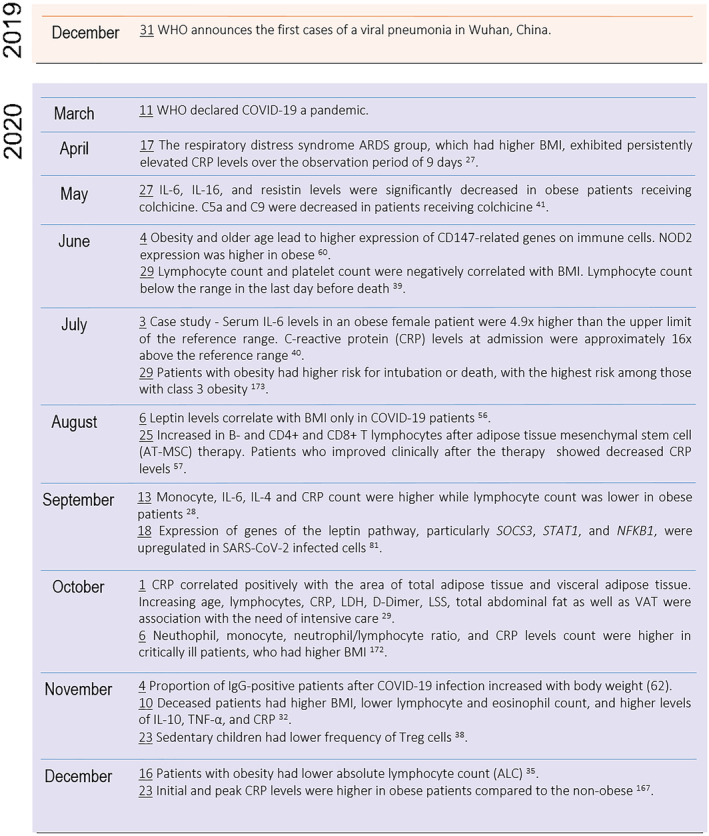
Timeline of the findings on the interaction among COVID‐19, obesity, and the immune system in 2019 and 2020. The results demonstrated were summarized in Tables 2, 3, 4. The date of each result and event is underlined and corresponds to the date of its first appearance in the databases

**FIGURE 5:**
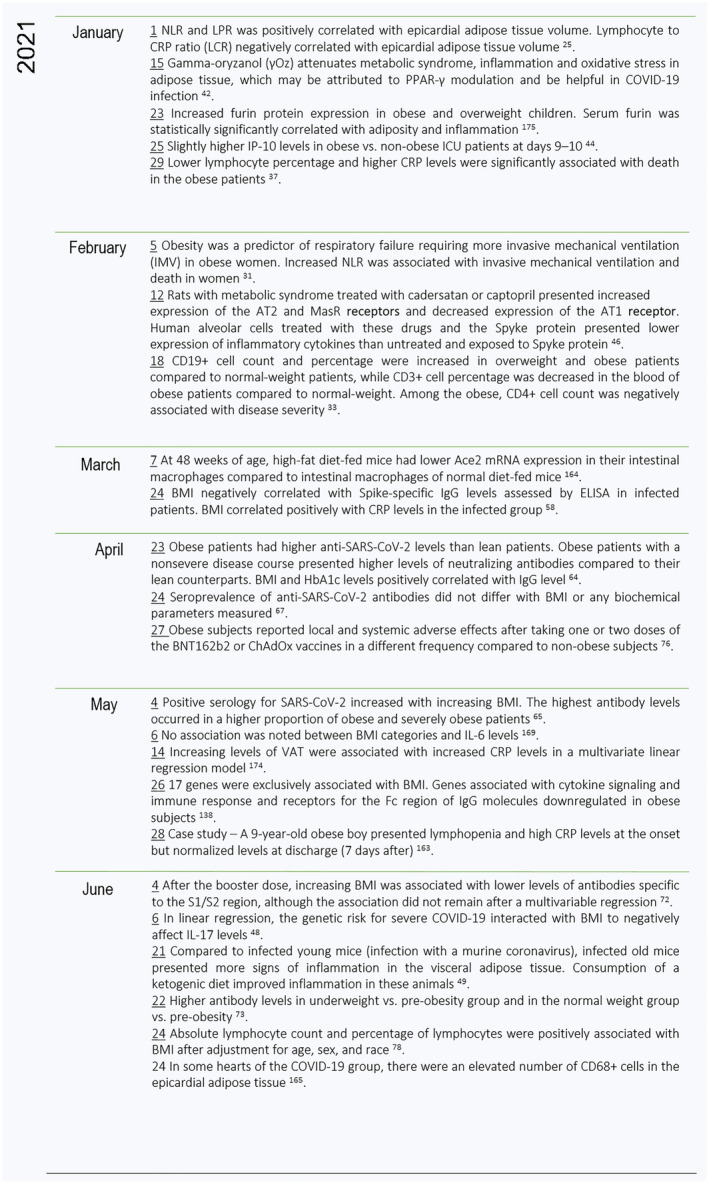
Timeline of the findings on the interaction among COVID‐19, obesity, and the immune system in the first semester of 2021. The results demonstrated were summarized in Tables 2, 3, 4. The date of each result and event is underlined and corresponds to the date of its first appearance in the databases

**FIGURE 6:**
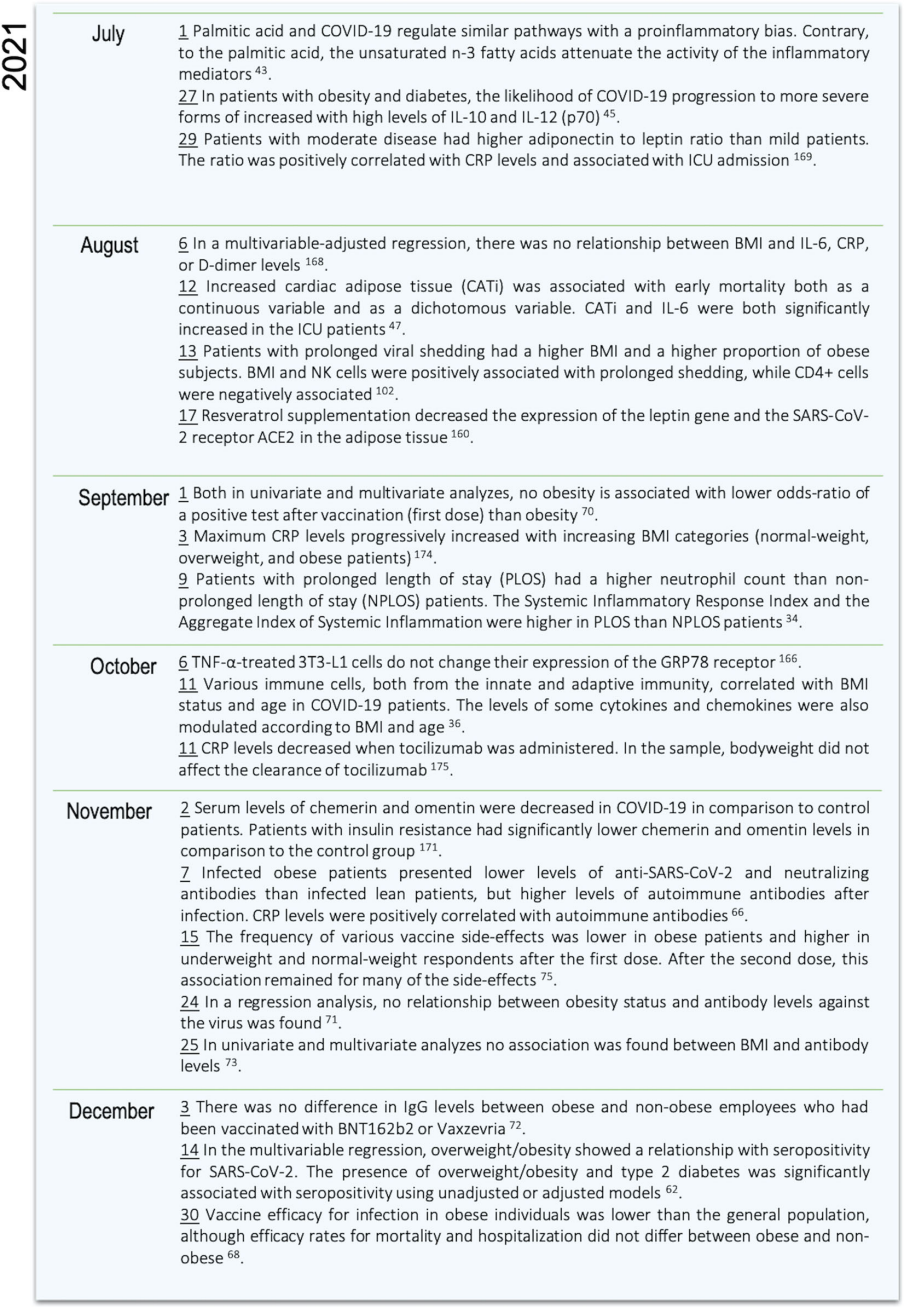
Timeline of the findings on the interaction among COVID‐19, obesity, and the immune system in the second semester of 2021. The results demonstrated were summarized in Tables 2, 3, 4. The date of each result and event is underlined and corresponds to the date of its first appearance in the databases

The present scoping review analyzed the studies and their results comprising the association between COVID‐19, adiposity, and the branches of the immune system with a focus on answering the primary research question. Overall, adiposity was found to be related to an elevated population of monocytes and neutrophils with a concomitantly lower lymphocyte count in COVID‐19 patients as compared to patients with normal weight. Cytokine and adipokine levels, the CRP inflammatory marker, and antibodies were also observed to differ according to BMI in COVID‐19‐positive patients. Moreover, some pathways seem to be differentially regulated by obesity in the context of SARS‐CoV‐2 infection. Adipose tissue plays a role in the development of the immunological alteration, given the correlation of specific depots with immune markers. Furthermore, vaccines are safe and effective in the population with obesity, although one of the studies included in the present review reported slightly lower efficacy, and two others observed different frequency of side effects in the group with obesity as compared to normal‐weight individuals. These findings suggest that the inflammatory background and the immune dysregulation patients with obesity face may sum to those observed in COVID‐19, and adiposity may influence the immune course of the disease as well as the immune response to vaccines. Figures 4, 5, 6 summarize the immunological findings in the relationship between COVID‐19 and obesity 2 years after the first public announcement by the WHO.

### 4.1 Immune cells

Considering the findings on immune cells, the NLR was higher in women, who had higher BMI than men, and was related to the epicardial adipose tissue volume.^25^, ^31^ However, based solely on the studies included in the present review, it is not possible to conclude that adiposity is directly associated with the NLR, as one of the included studies failed to observe any association between NLR and BMI, and another only pointed to an indirect relationship mediated by the length of hospital stay.^30^, ^34^ A relevant insight, however, may come from the studies analyzing neutrophil and lymphocyte counts. While neutrophils seem to be positively related to BMI levels,^26^, ^29^, ^33^, ^34^ lymphocytes are often lowered in patients with obesity.^26^, ^28^, ^29^, ^32^, ^35^, ^37^, ^39^, ^43^ Importantly, the adiposity profile and specific adipose tissue depots, such as the epicardial adipose tissue, may be more accurately related to NLR, as they discriminate adiposity better than the BMI.^25^ Indeed, it was observed that the NLR was associated with adiposity markers, particularly waist‐to‐height ratio and waist‐to‐hip ratio, although no difference was noted between patients with obesity and individuals without obesity.^97^ Moreover, the NLR is associated with fatty liver disease and insulin resistance, which are common in patients with obesity and may aggravate COVID‐19.^98^, ^99^ Thus, although BMI is probably not associated with higher NLR, adiposity levels probably are, and this is consistent with the data reporting worse outcomes in patients with obesity. Moreover, the higher NLR may be guided by the neutrophilia and lymphopenia these patients often present.

Neutrophilia, in particular, is a common feature of COVID‐19. It is related to the formation of neutrophil extracellular traps and possibly to the vascular and respiratory complications observed in the disease.^100^, ^101^, ^102^, ^103^ Patients with obesity often present higher neutrophil counts than normal‐weight individuals, and adipose tissue is enriched in those cells.^99^, ^104^, ^105^, ^106^ Importantly, neutrophils may play an important role in sustaining the inflammatory milieu in obesity, thus contributing to COVID‐19 severity as there is an upregulation of genes involved in neutrophil activation, such as myeloperoxidase (*MPO*) and neutrophil elastase (*ELANE*), in patients with obesity.^106^

Regarding the innate cell count, there are reports of monocytes/macrophages, eosinophils, basophils, NK, and dendritic cells in patients with obesity. Monocytes and macrophages appear to be associated with adiposity in patients with obesity and COVID‐19, with CD14^+^CXCR6^+^ cells positively correlated with the elevated BMI and CD68^+^ cells in the epicardial adipose tissue.^28^ The phenotype of monocytes and macrophages is, arguably, of higher relevance than the absolute count since healthy, mild, and severe patients may not differ in the absolute count yet differ in morphological and expression profiles of these cells.^107^, ^108^, ^109^ Nevertheless, the difference observed in monocytes may be due to obesity, as a higher cell count was observed in patients with obesity. Moreover, important differences were reported between patients with obesity and individuals without obesity in the classical, non‐classical, and intermediate compartments.^110^, ^111^ In this regard, the positive correlation between CD14^+^CXCR6^+^ cells and BMI in young infected patients is illustrative, given that CXCR6 is a lung homing marker and cells bearing this marker are involved in cardiac inflammation and remodeling, thus suggesting that these cells play an important role in the disease.^112^

While eosinophils, total, and myeloid dendritic cells were negatively associated with BMI in infected patients, basophils and NK cells were positively associated with BMI and prolonged viral shedding, respectively. Although it could not be ascertained whether these findings are directly related to BMI or to the severity of the disease – given that few studies have considered patients with obesity –, the literature reports important changes in the blood count and phenotype of these cells in cases involving COVID‐19 and obesity.^113^, ^114^, ^115^, ^116^ Importantly, these cells are susceptible to the cytokine milieu and direct effects of the virus, each having particular courses depending on disease severity and stage. As the studies reporting the association of these cells with obesity in COVID‐19 have a cross‐sectional design, it is possible that the changes observed reflect the disease stage when collection was performed. Eosinophils, for example, follow a particular pattern over time in the infection and in accordance with disease severity.^113^

The lymphocyte count was the main parameter assessed when considering the adaptive immunity. As mentioned, lymphopenia is a common finding in COVID‐19 cases. Following this trend, most studies reported a lower lymphocyte count in patients with obesity as compared to individuals without obesity. Interestingly, non‐infected individuals with obesity usually have a higher lymphocyte count than the normal‐weight controls.^117^, ^118^ However, when it comes to disease severity, obesity is a well‐known risk factor. Indeed, severe patients seem to have a different adaptive immune response than patients with milder ones, with a lower lymphocyte count and a delayed and considerably weaker T cell response, which may explain the findings presented here.^9^, ^119^

The phenotypic characterization of lymphocytes was also performed in some studies and may be insightful for understanding disease pathogenesis and the immunological nuances of the interaction between obesity and COVID‐19. Lower CD4^+^ T cell count in the blood was associated with disease severity and prolonged viral shedding, whereas lower levels of physical activity and higher BMI were associated with lower frequency of CD4^+^ Treg cells.^33^, ^38^, ^42^ These results corroborate the literature showing circulating CD4^+^ being affected by the infection and negatively associated with its severity and recovery.^120^, ^121^ However, the mechanism for this effect, as well as for the exacerbated lymphopenia in patients with obesity, has not yet been clearly identified but may be the result of the viral attack on these cells, cell death following activation, or redistribution and localization to specific body compartments.^120^ The result on Treg cells indicates that the higher BMI and the physical inactivity characteristic of the present pandemic are related to a higher inflammatory background, which could be of great importance for the clinical course of treatment in patients with obesity.

Despite these results, an important feature that was largely overlooked in the studies is the further characterization of the subsets of lymphocytes beyond CD4^+^ T cells. In general, there is an upregulation of both Th1 and Th17 profiles, including their characteristic cytokines, while Th2 and Treg profiles and their cytokines are downregulated in obesity.^122^, ^123^, ^124^, ^125^ The Treg/Th17 ratio is decreased in obesity, and this is related to metabolic health.^126^, ^127^ Severe COVID‐19, in turn, provokes dampened Th1 and Treg responses, while promoting the Th17 profile.^128^, ^129^, ^130^ Similar to obesity, the IL‐17/IL‐10 ratio is increased in COVID‐19 cases, even in deceased patients, as compared to those who improved.^130^ The cytokine milieu in these changes plays an important role. As will be discussed in the next section, IL‐5 participates in the event of cytokine storm, is often increased in obesity, and skews the polarization to a Th17 profile and away from the Treg profile.^131^ Interestingly, TGF‐β, a cytokine that along with IL‐6 is important for the Th17 development, is more frequently expressed in the leukocytes of children with obesity and has an inverse relationship with IL‐10 in neutrophils of children with obesity.^132^, ^133^ In this regard, it can be argued that the skewed pattern of CD4^+^ T cell subsets and their characteristic cytokines, which are already established in obesity, is further deepened in COVID‐19, which is a factor for greater severity and a higher mortality rate in patients facing the double burden of obesity and COVID‐19.

One study among the articles found in the present review reported a positive correlation between CD4^+^ central memory T cells and BMI in infected aged patients, while CD8^+^ cells secreting IFN‐γ correlated negatively with BMI in infected young patients.^36^ Contrary to total CD4^+^ cells, which drop sharply in the infection, memory CD4^+^ cells are effectively formed in COVID‐19 patients and readily stimulated in vitro.^134^ Like CD4^+^ cells, the CD8^+^ cell count is negatively associated with the severity of infection.^121^ Thus, the negative correlation found between CD8^+^ cells secreting IFN‐γ and BMI may be reflective of a dampened antiviral response in individuals with obesity. However, it is puzzling that these correlations were observed only in aged or young individuals. Nonetheless, important differences had already been noted in the adaptive immune response when age was considered.^135^ Still, the adipose tissue may possess an interesting therapeutic potential as the transfer of mesenchymal stromal cells from the adipose tissue of a healthy donor to severe patients resulted in a better outcome in most of the treated cases, accompanied by increased levels of B, CD4^+^, and CD8^+^ T cells.^41^ From these data, it can be hypothesized that the normal‐weight, non‐inflamed adipose tissue acts as a reservoir of lymphocytes that counterbalance the deleterious effects of the infection, while the adipose tissue of patients with obesity does not possess this potential.

In addition to the lymphocyte count, adiposity in the form of epicardial adipose tissue volume was related to the lymphocyte‐to‐CRP (LCR) and platelet‐to‐lymphocyte ratios. The LCR was negatively correlated with the epicardial adipose tissue volume, while the PLR was positively correlated with the same parameter. Both findings have been associated with a poor clinical status and outcome. A lower LCR is associated with an unfavorable outcome and higher BMI.^136^, ^137^ As discussed below, CRP is effectively elicited by IL‐6, which is often increased in obesity, thus explaining the lower ratio found in patients with obesity. Higher PLR is significantly associated with positive SARS‐CoV‐2 infection and severe disease, although this finding is not unanimous in the literature.^138^, ^139^, ^140^ Importantly, coagulation disorders are concerning events in COVID‐19. Indeed, platelet count – thrombocytopenia, particularly – and coagulation biomarkers, such as D‐dimer, prothrombin time, and fibrin degradation products are related to disease severity and disseminated intravascular coagulation, and the mechanism behind them may be the inflammatory alterations in cell composition and cytokine milieu, particularly lymphopenia, that patients with obesity affected by COVID‐19 face.^141^, ^142^, ^143^ Taken together, these results suggest that obesity exacerbates the inflammatory state in COVID‐19, and this is likely to play a major role in coagulatory disorders.

### 4.2 Immune molecules and pathways

The reports on immune molecules focused on the levels of cytokines/adipokines, CRP, and antibodies, but there were also important reports on immune receptors, complement proteins, lipid mediators, and the enzyme furin. The preference for the study of cytokines/adipokines and CRP levels may be related to the predictive value of these markers for complications in COVID‐19.^119^, ^144^ The dysregulated levels of cytokines are of great concern due to the cytokine storm severe patients may face.^145^ As in the case of other immune components reviewed here, it is not possible to present a clear scenario describing the involvement of these cytokines at the interface between obesity and COVID‐19 due to the plethora of confounding factors in each study. These factors include the fact that patients with obesity have a higher disease severity – and comparisons are often made with patients without obesity who did not have a similar disease course –, the definition of obesity used in each study, the co‐occurrence of other comorbidities that may mask the results, the limited metabolic characterization of patients with obesity, and the small sample size of many studies. The pandemic itself exacerbated various socioeconomical and biological factors – which are also important to account for in future analyses – that worsen the burden of obesity on the general population.^146^ Nonetheless, a trend can be observed toward higher levels of inflammatory molecules and activation of inflammatory pathways in patients with obesity and COVID‐19.

The most commonly studied cytokine in the studies included in this review is interleukin 6 (IL‐6), with some studies reporting higher IL‐6 levels in patients with obesity. IL‐6 is usually elevated in obesity, with the adipose tissue being a major contributor to its circulating levels, as adipocytes and immune cells of the adipose tissue are important sources.^147^, ^148^ In COVID‐19‐positive patients, IL‐6 is an important inflammatory cytokine elicited by the inflammatory stimulus and is one of the main cytokines in the event of a cytokine storm, being correlated to an unfavorable clinical outcome.^144^, ^145^ The results of the IL‐6 receptor (IL‐6R) inhibitor tocilizumab on patients with obesity also highlight the importance of this cytokine as a key hotspot in the interaction between obesity and COVID‐19.^80^ IL‐10 and TNF‐α are also common, with studies showing mixed results. While IL‐10, an anti‐inflammatory cytokine, is usually lower in patients with obesity, TNF‐α, a proinflammatory cytokine, shows the opposite trend. Surprisingly, in COVID‐19, IL‐10 is often elevated in severe patients and is an important cytokine in the event of a cytokine storm and may have a pathological role to play in the disease.^57^, ^58^ These high levels of IL‐10 may be an attempt to reduce hyperinflammation and prevent tissue damage. However, the elevated production of IL‐10 and pro‐inflammatory cytokines, coupled with the observed association between higher IL‐10 levels and disease severity, suggests that IL‐10 is either failing to adequately suppress inflammation or acting in a way that deviates from its traditional role as an anti‐inflammatory molecule.^149^ TNF‐α is also elevated and has been shown to have a strong predictive value for disease severity and death, being involved in tissue destruction and inflammatory cell activation in COVID‐19.^56^, ^150^, ^151^

Among the adipokines, leptin levels were elevated in infected patients, although it may be due to their higher BMI.^62^ Lower leptin levels were observed in an animal model supplemented with an anti‐inflammatory and antioxidant substance, revealing that this molecule may be downregulated by anti‐inflammatory agents.^48^ Due to its immunometabolic roles, leptin plays a major inflammatory role, triggering inflammatory responses.^152^ In fact, one of the studies summarized in the present scoping review revealed that key inflammatory genes in the leptin pathway, *SOCS3, STAT1*, *NFKB1*, and *IL1B*, are upregulated in infected human epithelial cells.^66^ Moreover, deceased patients with obesity as one of the comorbidities presented upregulation of genes in the leptin pathway in lung tissue samples.^74^ It has been suggested that leptin may be a facilitator of acute pulmonary inflammation, given its immune role and increased angiotensin II, which promotes cardiorespiratory derangements.^62^ Taken together, these results point to leptin, in addition to IL‐6, as an important inflammatory molecule produced by the adipose tissue that may be responsible for triggering detrimental effects in COVID‐19.

There is a trend in the studies denoting higher CRP levels in patients with obesity and a positive association with adiposity. CRP is often higher in patients with obesity as compared to normal‐weight controls and is associated with the low‐grade inflammation characteristic of obesity.^111^, ^132^, ^153^ Blood CRP concentration in COVID‐19 cases is a common biomarker for adverse outcomes.^154^ As discussed above, IL‐6 is a key player in COVID‐19 pathogenesis and is often elevated in infected patients with obesity as compared to those without obesity. This cytokine is a potent transcriptional stimulator of CRP in the liver.^155^ Therefore, in the case of an infected patient with obesity, there may be a summation of both conditions for elevated CRP levels.

The reviewed studies reveal that obesity is positively associated with the expression of proinflammatory receptors and pathways, and this may be important in COVID‐19. In an animal model of metabolic syndrome, a higher expression of AT1 and MasR receptors was observed, which mediate anti‐inflammatory and cardioprotective actions, and the lower expression of the proinflammatory receptor AT2 after the administration of candersatan and captopril.^52^ These results suggest that metabolically unhealthy individuals, including those with obesity, may suffer the cardiorespiratory and inflammatory consequences of the dysregulation of the renin‐angiotensin system. Indeed, patients with obesity present higher levels of the vasopressor molecule angiotensin II, which may exacerbate clotting formation, inflammation, and the cardiac and pulmonary damage seen in COVID‐19 cases.^156^, ^157^ The higher NOD2 expression in patients with obesity likely indicates heightened surveillance to bacteria, as this receptor recognizes fragments of bacterial peptidoglycan, such as muramyl dipeptide, and this is relevant in complicated infections where bacteria are involved.^158^ The higher expression of *NFKB1* in the leptin pathway using expression data collected from non‐infected epithelial cells once again indicates that leptin, which is directly associated with adiposity, is an important inflammatory mediator.^66^

Signs of complement activation were suggested to have a relationship with adiposity. The patients with obesity taking colchicine had lower levels of C5a and C9 as compared to patients with obesity under placebo, and complement activation was observed in the endothelium of the subcutaneous adipose tissue associated with the presence of the virus. These data suggest that patients with obesity may be more prone to complement activation and that it is linked to subcutaneous manifestations of COVID‐19. SARS‐CoV‐2 is indeed capable of exacerbating complement activation via the classical and the lectin pathways, which is associated with disease severity due to endothelial damage and thrombi formation in multiple organs.^159^, ^160^

The data on antibodies are puzzling, as different studies have reported decreased, increased, or similar titers or seroprevalence between patients with and without obesity and COVID‐19. Several factors may be affecting these relationships, such as disease severity, study design, and specific antibodies analyzed. Disease severity, in particular, may drive a positive association between antibody levels and adiposity, as patients with obesity have a higher prevalence of worse clinical outcomes. For instance, hospitalized patients and children with multisystem inflammatory syndrome present higher IgG levels directed to the S protein as compared to those patients with a less severe disease course.^10^, ^161^, ^162^ The data on antibodies directed to specific viral proteins and aimed at neutralizing antibodies, though more informative than total IgG, are also difficult to interpret. While anti‐Spike IgG was shown to have an inverse correlation with BMI,^63^ anti‐nucleoprotein IgG showed a positive correlation with the same parameter only in young patients. These results indicate that some viral antigens present differing degrees of immunogenicity for the immune system, and this may be dependent on both adiposity and age. There is a report indicating higher levels of neutralizing antibodies in non‐severe patients with obesity as compared to non‐severe lean patients and higher total anti‐SARS‐CoV‐2 antibodies in patients with obesity. Another study, however, has shown a lower frequency of individuals with obesity with neutralizing antibodies and lower anti‐SARS‐CoV‐2 titers, while also reporting higher antibody levels directed to self‐molecules,^78^ which may reflect the higher cell death that occurs in the adipose tissue of patients with obesity. These findings suggest that the higher antibody levels present in patients with obesity do not relate to disease protection but may mediate tissue damage with part of these antibodies directed to self‐tissues. Regardless of the discrepancies, it can be argued that individuals with obesity have a particular dynamic of B cell activation and humoral immune response, which has already been demonstrated in animal models of obesity.^163^, ^164^

### 4.3 Vaccines

Finally, vaccines are considered to be safe and effective in patients with obesity, based on data from vaccine studies in this population, although they may be marginally less effective for protection against infection and may induce different frequencies of side effects as compared to the normal‐weight population. Although these data have not yet been replicated, they suggest the immune system of patients with obesity responds to vaccines in specific ways. The lower efficacy for preventing infection in patients with obesity receiving the BNT162b2, in particular, may be reflected in other studies included in this review, which reported lower antibody levels in health‐care staff with obesity after receiving the first and second doses of the same vaccine.^88^, ^93^, ^94^ However, one study observed no difference between individuals with and without obesity after receiving the vaccine, yet noted a higher risk of infection in those with obesity after receiving the first dose of BNT162b2, ChAdOx‐1 nCoV‐19, or mRNA‐1273.^87^, ^89^, ^90^, ^93^, ^94^ Multiple immune mechanisms may be backing these results. The results of the present review clearly demonstrate that the innate and adaptive immune responses are altered in obesity. One study showed that BMI in COVID‐19 patients correlated with dendritic cells and central memory CD4^+^ T cells, two important cell types in the immune response following immunization.^36^ Considering the antibody response specifically, individuals with obesity may present a unique humoral response with higher antibody titers but lower protection, as discussed above. Previous reports on the vaccination of patients with obesity have also observed mixed results, with BMI either negatively associated with efficacy and antibody levels or having no relationship with these parameters.^165^, ^166^, ^167^ The response to vaccines is multifactorial, with variables such as age, genetic background, and vaccine technology likely affecting the outcome.

Some remarks need to be taken into consideration in light of the reviewed studies, the first one being the definition of obesity. In some studies, obesity or normal weight was defined by adiposity measures or different BMI setpoints, although most studies classified BMI categories according to the WHO criteria. BMI alone may not be the best tool to assess adiposity and, consequently, the association between obesity and COVID‐19. Most of the studies assessed obesity through BMI. However, this approach may shadow some important aspects of the relationship between obesity and COVID‐19. Although BMI serves as an indirect indicator of adiposity in a population, and the association between BMI and COVID‐19 severity appears to be a V‐shaped curve with severity increasing almost linearly in overweight and individuals with obesity (BMI > 25 kg/m^2^),^168^ direct measures of adiposity and especially visceral adiposity, provide a more precise indicator of the association behind obesity and COVID‐19.^75^, ^169^ Moreover, other variables, such as disease severity, age, and comorbidities are usually present and not controlled, which may explain the multiplicity of results found in certain topics. Another limitation is the transversal nature of most of the studies, which did not provide a clear picture of how patients with obesity evolve in immunological terms. Lastly, the phenotypes of innate or adaptive immune cells were often not considered when analyzed. Some cell types, such as monocytes and lymphocytes, can assume various forms that may possess contradictory actions that could not be captured given that only the cell count was reported.

### 4.4 Perspectives

Knowledge of COVID‐19 has advanced in various areas since the WHO declared it a pandemic, including those related to the pathogenesis of the disease and the immunopathology that accompanies it. This growing body of information has been crucial to confronting the pandemic, vaccines arguably being the pinnacle of applied knowledge associated with immunity against the virus. It has also extended to diseases that are important risk factors for COVID‐19 infection and severity, such as obesity.

Although important results have shed light on the relationship between obesity, COVID‐19, and the immune system, there are major gaps that still need to be addressed through future research. One of them is the course of infection in patients with obesity. The patients with obesity are well‐known to have a low‐grade inflammatory state, but it is not yet known how it affects the immune response against the virus. For example, in influenza A viruses, which include the H1N1 virus responsible for the 2009 pandemic, it was shown that important changes occur in the innate and adaptive immune systems, as well as longer viral shedding, both in humans with obesity and in animal models.^170^ Another important indicator that should be addressed is how metabolically healthy patients with obesity deal with the disease as compared to metabolically unhealthy patients with obesity from an immune perspective, considering these patients have different inflammatory profiles.^171^ Attention should also be paid to the definition of the sample, as there is a continuum ranging from patients without any metabolic compromise to those presenting all symptoms of the metabolic syndrome.^172^

The phenotypic characterization of cell populations may also be highly relevant to understanding how obesity affects and is affected by COVID‐19 infection. In addition to the cell count, obesity in both young and adult populations is associated with changes in the expression of cytokines, signaling the presence of molecules and receptors in the peripheral blood and adipose tissue.^111^, ^132^, ^133^, ^173^ A broader characterization in the blood, adipose tissue, and airways could highlight several aspects of the disease in patients with obesity. Moreover, adipokines may be key players in the disease in the context of patients with obesity. Leptin has been explored in some studies, but there are other hormones, such as adiponectin, resistin, and visfatin, that are also highly relevant due to their metabolic and immune influences. Adipose tissue, specifically, may be a key tissue for immune regulation of the disease in both normal weight and obesity, given the increased ACE‐2 expression due to a high‐fat diet in this tissue and the role of adipocytes as producers of inflammatory molecules and antigen‐presenting cells.^174^, ^175^

As one of the key elements in the immune response elicited by vaccines, the antibody response was shown to be more persistent in patients with obesity.^82^

Cellular immunity in obesity, however, is not known despite its highly important function following vaccine administration. Although one report suggests a positive correlation between CD4^+^ central memory cells and BMI in aged patients,^36^ it would be interesting to know whether memory B and T cells are effectively generated following the infection and, especially, after vaccination in patients with obesity. The effects of vaccination in the population with obesity also call for additional clarification. Although vaccines are highly effective and safe for this population, the studies included in this scoping review suggest a slightly discrepant post‐vaccination immune response in obesity. Longitudinal data on cellular and serologic variables would provide a better picture of this relationship.

In addition to vaccines, the efficacy of pharmacological treatments for COVID‐19 in the population with obesity warrants additional research. In this regard, the results for tocilizumab and colchicine have been promising. Both drugs have proven to be effective in reducing inflammation through various pathways: they reduced CRP levels in patients with obesity, while colchicine was also effective in reducing IL‐6, IL‐16, resistin, complement proteins C5a and C9, and the activity of the COX‐2 enzyme.^40^, ^47^

## 5 CONCLUDING REMARKS

More than six million people have died worldwide from COVID‐19, with many of these deaths in patients with comorbidities, such as obesity. Obesity is a major risk factor for disease severity and death and is associated with a low‐grade inflammatory state, which affects the entire body and all the immune branches. In the context of COVID‐19 immune response, obesity is associated with several differences as compared to normal‐weight individuals that signify a higher inflammatory background in the former (Figures 2 and 3). Despite the extensive timeline comprising studies made over the last 2 years, there are a number of considerations that have yet to be addressed relative to the COVID‐19 pandemic. It remains unclear whether these differences are due to discrepancies in the care received or to molecular mechanisms that predispose certain individuals to develop long‐lasting symptoms. The impact of the novel strains on the development of long COVID‐19 and which patients will be most affected by them will also require further study. It is not yet known whether this is more prevalent in individuals with obesity and whether the inflammatory state is involved. The exact mechanism also remains unknown.

## CONFLICT OF INTEREST

The authors declare no conflict of interest exist.
